# Targeting Iron Regulatory Protein 2 in Tumor-associated Macrophages Activates Antitumor Immunity in NSCLC via Modulating SphK1-PD-L1 Axis

**DOI:** 10.7150/ijbs.130591

**Published:** 2026-09-03

**Authors:** Jiawen Ding, Qinpei Cheng, Chen Cheng, Ying Mei, Liu Yang, Peilin Chen, Lei Zheng, Suhua Zhu, Kaikai Shen, Kuanyu Li, Tangfeng Lv

**Affiliations:** 1 Department of Respiratory and Critical Care Medicine, Jinling Hospital, Affiliated Hospital of Medical School, Nanjing University, Nanjing, China.; 2 Key Laboratory of Chinese Medicine Resource and Compound Prescription, Ministry of Education, Hubei University of Chinese Medicine, Wuhan, China; Center of Traditional Chinese Medicine Modernization for Liver Diseases, Hubei University of Chinese Medicine, Wuhan, China; Hubei Shizhen Laboratory, Wuhan, China.; 3 Jinling Clinical Medical College, Nanjing University of Chinese Medicine, China.; 4 Department of Pathology, Jiangsu Province Geriatric Hospital, Geriatric Hospital of Nanjing Medical University, Nanjing 210009, Jiangsu Province, China.; 5 Department of General Surgery, Vascular Surgery, Qilu Hospital of Shandong University, Jinan, Shandong, China.; 6 State Key Laboratory of Pharmaceutical Biotechnology, Jiangsu Key Laboratory of Molecular Medicine, Medical School, Nanjing University, Nanjing 210093, China.

**Keywords:** NSCLC, TAMs, IRP2, SphK1, PD-L1

## Abstract

Non-small cell lung cancer (NSCLC) is the leading cause of cancer-related mortality worldwide. As a predominant immune cell population in the tumor microenvironment (TME), tumor-associated macrophages (TAMs) play a critical role in tumor progression and resistance to immune checkpoint inhibitors (ICIs). TAMs can promote either antitumor immunity or immunosuppression depending on their polarization status. Iron regulatory protein 2 (IRP2) is a key regulator of intracellular iron homeostasis and has been reported to be upregulated in several tumor types. However, the role of IRP2 in the TME remains unclear. This study revealed the previously unrecognized role of IRP2 in NSCLC progression and explored its potential as a TAM-directed therapeutic target linked to programmed death-ligand 1 (PD-L1) regulation. We found that IRP2 skewed TAMs toward an immunosuppressive state that promoted tumor cell proliferation and migration, and limited apoptosis. Furthermore, IRP2 may sustain PD-L1 expression in TAMs by limiting sphingosine kinase 1 (SphK1)-associated lysosomal degradation. In conclusion, our findings identify IRP2-dependent maintenance of the immunosuppressive TAM phenotype as a promising therapeutic target in NSCLC.

## Introduction

Lung cancer remains the leading cause of cancer mortality worldwide, with non-small cell lung cancer (NSCLC) accounting for the majority of cases^1^-^3^. Lung cancer treatment has evolved from cytotoxic chemotherapy to molecularly targeted therapy and immunotherapy. In particular, immune checkpoint inhibitors (ICIs) have transformed the treatment landscape^4^. ICIs targeting the programmed death-1 (PD-1)/programmed death-ligand 1 (PD-L1) axis have transformed NSCLC treatment and have achieved a major therapeutic breakthrough by inducing durable clinical responses in a subset of patients^5^, ^6^, yet both primary and acquired resistance remain common and limit overall benefit^7^, ^8^.

Many studies have shown that tumor-associated macrophages (TAMs) constitute the predominant population and act as key drivers of immune evasion within the tumor microenvironment (TME)^9^-^11^. TAMs are highly plastic, polarizing into classically activated (M1) or alternatively activated (M2) phenotypes in response to the stimuli of the TME. M1 TAMs contribute to anti-tumor immunity by pro-inflammatory responses and antigen presentation, whereas M2 TAMs facilitate tumor progression through enhanced angiogenesis, extracellular matrix remodeling, and inhibition of cytotoxic lymphocyte functions^12^, ^13^. The protumoral TAMs suppress cytotoxic T lymphocyte (CTL) responses and are associated with reduced ICI efficacy^14^. Notably, TAMs are a major source of PD-L1 in the TME, and PD-L1 expressed on TAMs represents a critical mechanism underlying CTL dysfunction and resistance to PD-1/PD-L1 blockade^15^, ^16^. Previous studies have indicated that the expression of PD-L1 on TAMs is regulated by transcription factors (e.g., STAT3) and post-translational modifications (e.g., glycosylation, ubiquitination)^17^, ^18^. Further clarification of the PD-L1 regulatory pathways in TAMs remains imperative, as this is crucial for addressing the key knowledge gap in immunotherapy resistance, and thereby improving the therapeutic outcomes of NSCLC.

Iron metabolism critically regulates tumor progression and antitumor immunity^19^-^21^. As major iron-handling cells within the TME, TAMs exhibit polarization-associated differences in iron handling, and their polarization states further shape inflammatory responses, antigen-presenting capacity, and immune regulation. In general, M1 TAMs typically adopt an iron-sequestering phenotype that may restrict iron availability to tumor cells, whereas M2 TAMs are more prone to release iron, potentially supporting tumor progression^22^. Therefore, defining how iron metabolic regulators shape TAM function is important for understanding the immunosuppressive mechanisms within the tumor immune microenvironment.

Iron regulatory protein 2 (IRP2) is a key post-transcriptional regulator of iron homeostasis. Beyond its role in controlling iron uptake, storage and utilization, IRP2 also regulates iron-dependent cellular metabolic programs, including mitochondrial respiration and glycolytic activity. These metabolic processes are highly relevant to macrophage polarization, antigen presentation, and immunoregulatory activity^23^-^28^. Previous work revealed that IRP2 is essential for maintaining macrophage immune responses under inflammatory conditions, partly by preserving lysosomal biogenesis and function^29^. These findings indicate that IRP2 may serve as a molecular bridge connecting iron metabolism, lysosomal activity, and macrophage immune regulation. However, the mechanism by which IRP2 influences TAM-mediated immune evasion in NSCLC remains unclear.

PD-L1 abundance is regulated not only at the transcriptional level but also through post-translational mechanisms that control its protein stability, including intracellular trafficking and lysosomal degradation. Given that IRP2 has been found to regulate lysosomal function in macrophages, we sought to investigate whether IRP2 in TAMs drives immune evasion via lysosomal mechanisms. Sphingosine kinase 1 (SphK1) was found to localize mainly to lysosomes in brown adipose tissue, and genetic deletion of *Sphk1* impaired lysosomal biogenesis and function^30^. In lung adenocarcinoma (LUAD), elevated SphK1 expression correlates with poor survival and constitutes both an independent prognostic factor and a tractable therapeutic target^31^-^33^. Nevertheless, it remains unclear whether SphK1 provides a functional link between IRP2-dependent metabolic regulation, lysosomal PD-L1 stability and TAM-mediated immune suppression in NSCLC.

Based on these observations, we hypothesized that IRP2 sustains PD-L1 expression on TAMs by limiting SphK1-associated lysosomal degradation. We further investigated the effects of IRP2 on the immunoregulatory functions of TAMs and the therapeutic response to PD-1 blockade. Our work sought to characterize the IRP2-SphK1 axis as a novel mechanism of PD-L1 regulation and identify a potential therapeutic target to enhance immunotherapy efficacy in NSCLC.

## Materials and Methods

### Sample collection

Five pairs of lung adenocarcinoma tissues and corresponding paratumoral tissues were obtained from patients undergoing pulmonary resection at Jinling Hospital Affiliated to Nanjing University Medical School. Written informed consent was obtained from all participants, and the study was approved by the Ethical Committee and Institutional Review Board of Jinling Hospital (approval number: 2023DZGZR-028).

### Animals and tumor models

All animal experiments were approved by the Ethical Review Committee of Jinling Hospital (approval number: 2023JLHGZRDWLS-00030). The control C57BL/6* Irp2^loxp/loxp^* mice (Cyagen Biosciences, China) were bred with LysM-Cre mice to generate* Irp2^LysMCre^* mice. All animals used through the study were 8-12 weeks old mice on C57BL/6 background, and maintained under a constant 12h light/dark cycle with ad libitum access to water and standard chow with supplements. All animals received humane care.

For tumor establishment, 1.5 × 10^6^ Lewis lung carcinoma (LLC) cells suspended in 50 μL phosphate-buffered saline (PBS) were injected subcutaneously into the right flank of 8-10 weeks old male mice. Tumor size was measured every 2-3 days using calipers and calculated as tumor volume using the following formula: tumor volume (mm^3^) = (length × width^2^)/2. Between day 14 to 21 when the experimental endpoint was met or when the longest tumor diameter reached 20 mm, mice were anesthetized and sacrificed according to NIH guidelines. Tumors were excised, weighed, and kept in PBS on ice, then processed for flow cytometry analysis, fixed with paraformaldehyde, or frozen.

For *in vivo* pharmacological inhibition experiments, tumor-bearing mice were randomized into different treatment groups when tumor volumes reached 50-100 mm³. Chloroquine phosphate (CQ; MedChemExpress, USA) was freshly dissolved in sterile PBS while PF-543 (MedChemExpress, USA) was initially dissolved in dimethyl sulfoxide (DMSO) and further diluted in a solvent consisting of 40% PEG-300, 5% Tween 80 and sterile saline. Mice were treated with CQ 60 mg/kg or PF-543 20 mg/kg every other day by intraperitoneal injection. Mice in the control groups received an equal volume of the corresponding vehicle.

For checkpoint blockade, when tumor volumes reached 50-100 mm³, mice were randomized into different treatment groups. Mice were then treated with anti-PD-1 mAb (clone: RMP1-14, Bio X Cell, USA) at a dose of 200 μg per mouse or PBS by intraperitoneal injection twice a week. Tumor volume was measured every 3 days, and body weight was monitored throughout the experiment. Mice were euthanized on day 21 after tumor inoculation.

### Cell line

The LLC cell line was purchased from the Institute of Biochemistry and Cell Biology of the Chinese Academy of Sciences (Shanghai, China). LLC cells were cultured in DMEM supplemented with 10% fetal bovine serum (FBS), 1% streptomycin and penicillin, 1% glutamine, and 1% sodium pyruvate at 37°C and 5% CO₂.

### Bone marrow-derived cell culture and differentiation into macrophages

Bone marrow-derived cells (BMDC) were collected from the bilateral femurs and tibias of 4- to 5-week-old mice using PBS under sterile conditions. Single-cell suspensions were prepared and red blood cells (RBC) were lysed with RBC lysis buffer (Solarbio, China). After centrifugation, cells were seeded into 10-cm dishes and cultured in DMEM medium supplemented with 10% FBS. On the second day, the medium was supplemented with 50 ng/mL macrophage colony-stimulating factor (M-CSF; MedChemExpress, USA). Fresh medium containing 50 ng/mL M-CSF was added to the cell cultures on day 4, and the cells were cultured until day 7 to obtain bone marrow-derived macrophages (BMDMs) for further experiments.

### Cell treatments

To induce a TAM phenotype, BMDMs were cultured* in vitro* with LLC-conditioned medium (LLC-CM) for 48 h. For co-culture experiments, BMDMs were co-cultured with LLC cells for 48 h. To investigate the pathways involved in PD-L1 degradation associated with *Irp2* deficiency, TAMs were treated with 10 μM MG132 (MedChemExpress, USA) and 10 μM CQ for 4 h. To detect whether IRP2 affects SphK1, TAMs were treated with 5 μM PF-543 for 4 h. TAMs were treated with 100 nM phorbol 12-myristate 13-acetate (PMA; Sigma, USA) for 12 h to induce SphK1 upregulation and treated with exogenous 1 μM sphingosine-1-phosphate (S1P; MedChemExpress, USA) for 6 h to examine the role of SphK1 in lysosome-associated PD-L1 regulation.

### Hematoxylin and eosin (H&E) staining and immunohistochemistry (IHC) analysis

Mouse organ tissues were fixed in 4% buffered formalin and then paraffin-embedded. Paraffin-embedded samples were sectioned at a thickness of 4 micrometers and processed for H&E staining. For IHC staining of mouse tissues, sections were deparaffinized and subjected to antigen retrieval by heating in 10 mmol/L citrate buffer (pH 6.0). Endogenous peroxidase activity was blocked by treatment with 3% hydrogen peroxide. Sections were then washed in deionized water, rinsed in PBS, and blocked with 3% BSA (Biofroxx, Germany) in PBS at room temperature. Subsequently, sections were incubated with primary antibodies against F4/80 (Abcam, UK), Ki-67 (Proteintech, China), CD4 (CST, USA), CD8 (CST, USA), and Caspase3 (CST, USA) overnight at 4°C in a humidified chamber. After washing with PBS, sections were incubated with horseradish peroxidase (HRP)-conjugated secondary antibodies for 1 h. All the antibodies used are listed in Supplementary Table S1. Immunoreactivity was visualized using 3,3′-diaminobenzidine (DAB; Dako, Denmark) according to the manufacturer's instructions. The immune reaction was examined under a microscope (Olympus, Japan). To quantify positive cells, the number of positive cells in 20× fields was counted across an average of three sections per sample.

### Immunofluorescence analysis

Mouse subcutaneous tumors and paired human tumor and paratumoral tissues were prepared as frozen sections. For immunofluorescence staining of cultured cells, cells were seeded onto sterile coverslips. The samples were first fixed in 4% buffered formalin, permeabilized with or without Triton X-100 and blocked with bovine serum albumin (BSA). Subsequently, primary antibodies F4/80 (Abcam, UK), Caspase-3 (CST, USA), PD-L1 (Proteintech, China), LAMP1 (Abcam, UK), IRP2 (HUABIO, China), CD68 (Abcam, UK), Pan-CK (CST, USA), CTSB (Proteintech, China), and SphK1 (Proteintech, China) were applied and incubated overnight at 4°C. All the antibodies used are listed in Supplementary Table S1. On the second day, the samples were washed and incubated with Alexa Fluor 488 and Alexa Fluor 594-conjugated secondary antibodies (Invitrogen, USA) in the dark at 4°C for 1 h. The tissue sections were mounted with an anti-fade mounting medium containing 4',6-diamidino-2-phenylindole (DAPI; Beyotime, China) and imaged under confocal microscope (Olympus, Japan) and analyzed with NIH ImageJ software.

### Reverse transcription-quantitative polymerase chain reaction (RT-qPCR)

Total RNA was extracted using TRIzol reagent (Vazyme, China). The extracted RNA was then reverse-transcribed into cDNA using the HiScript III RT SuperMix for qPCR (Vazyme, China). RT-qPCR was performed to measure the mRNA expression levels of the genes using the ChamQ SYBR qPCR Master Mix (Vazyme, China). 18S rRNA was used as an internal control. The relative mRNA expression levels were calculated using the 2^^-ΔΔCt^ method. The primer sequences are provided in the Supplementary Table S4.

### Western blot analysis

Collected cells were washed with ice-cold PBS and lysed in lysis buffer. Protein concentrations in the lysates were measured using the standard Bradford assay. For Western blot, 15 to 25 µg of total protein from the cell lysates was separated by sodium dodecyl sulfate polyacrylamide gel electrophoresis (SDS-PAGE). The separated proteins were transferred from the gel to a nitrocellulose membrane. The membrane was then blocked with 5% non-fat milk in 0.1% Tris-buffered saline containing Tween 20 (TBST) buffer. Subsequently, the membrane was incubated with primary antibodies, followed by HRP-conjugated secondary antibodies. All the antibodies used are listed in Supplementary Table S1. Proteins were visualized using enhanced chemiluminescence and imaged with a digital Western blot imaging system (Tanon, China).

### Colony formation assay

LLC cells were seeded (1000 cells/well) in 6-well plates and incubated at 37 °C overnight. The following day, the upper chambers of Transwell inserts containing BMDMs were placed on the LLC cells according to the experimental requirements for co-culture. Then, cells were washed twice with PBS, fixed with paraformaldehyde, and stained with crystal violet solution. The plates were washed with water, dried and photographed.

### Scratch assay

Cell migration was assessed by scratch assay. The scratch assay was performed as previously described^34^. LLC cells were seeded in six-well plates. The following day when cells reached 80-90% confluence, a straight line was scratched across the monolayer using a 100-μL pipette tip. And then LLC cells were cultured with DMEM alone or co-cultured with *Irp2^loxp/loxp^* or* Irp2^LysMCre^* TAMs in the upper chambers of Transwell inserts. Taking pictures after scratching (0 h, 24 h and 48 h) and measuring the area, then the cell migration rate was calculated based on the above observation. The images of scratch area were captured by Nikon Eclipse TS100. To avoid the influence of cell proliferation on migration, the experiments were conducted under serum-free culture conditions.

### Flow cytometry detection of cell apoptosis

LLC cells were seeded in 12-well plates at a density 5 × 10^4^ per well. After culturing overnight, cells were incubated in the presence of *Irp2^loxp/loxp^* and *Irp2^LysMCre^* TAM-CM. The untreated cells were regarded as negative control. After being incubated for 24 h, cells were then collected, washed with PBS, and stained with 100 μL 1× Annexin V binding buffer containing Annexin V-FITC and PI followed by adding binding buffer to detect cell apoptosis with flow cytometry.

### Flow cytometry analysis

The resected mouse tumor was isolated and dissociated in a digestion mixture containing 125 μg/ml Liberase TL (Roche, Switzerland) and 100 U/ml DNase I (Roche, Switzerland). Single-cell suspension prepared from tumor tissues were pre-incubated with anti-CD16/32 monoclonal antibody (FcR-blocking, BD, USA). Dead cells were marked using Zombie NIR Fixable Viability Kit (BioLegend, USA). Surface antibodies were added to the cell suspension, and staining was performed at 4°C in the dark. Next, the cells were permeabilized using Transcription Factor Fixation/Permeabilization Working Solution (BD, USA) and then intracellular antibodies were added and staining was conducted. Data were collected using CytoFlex and analysis was performed with FlowJo software. All the fluorescently labeled antibodies used for staining are listed in Supplementary Table S2 and panel used for flow cytometry is listed in Supplementary Table S3. Flow cytometry gating strategies were established using isotype and fluorescence-minus-one (FMO) controls and are shown in Supplementary Figure S1.

### Quantitative lipidomics analysis

Quantitative lipidomics analysis was performed by Guangke Ande Biotechnology Co., Ltd. Cell samples were harvested, washed with ice-cold PBS, and processed on dry ice. Lipids were extracted using an internal-standard-containing extraction solution by vortexing, homogenization, and sonication in an ice-water bath. After centrifugation, the supernatants were collected, dried under vacuum at 37°C, and reconstituted in resuspension buffer. The reconstituted samples were centrifuged again, and the resulting supernatants were transferred to glass vials for LC-MS/MS analysis. Equal amounts of each sample were pooled to prepare a quality control (QC) sample. Chromatographic separation was performed using a Nexera LC-40 UHPLC system, and lipid detection was conducted on a SCIEX Triple Quad™ 7500 mass spectrometer in multiple reaction monitoring mode. Data acquisition and quantitative analysis were performed using SCIEX OS and BioBud software.

### RNA sequencing

The BMDMs were treated with DMEM or LLC-CM for 48 h. The culture medium was completely removed and 1 mL TRIzol was added. The cells were lysed by repeated pipetting and the cell lysates in TRIzol were sent to Novogene Co., Ltd. (China) for RNA extraction and sequencing analysis.

### Statistics

Data were presented as the mean value ± standard error of the mean (SEM). All experiments were repeated at least three times independently. Student's t-test, one-way ANOVA and two-way ANOVA were performed using GraphPad Prism 8, as appropriate. Effect sizes were calculated using GraphPad Prism 11 from the same raw datasets used for the corresponding statistical analyses.* P* values are indicated in the figures, and significance was considered at *P* < 0.05.

## Results

### High IRP2 expression is associated with poor prognosis in NSCLC patients

To investigate the role of IRP2 in lung cancer, proteomics data from The University of Alabama at Birmingham CANcer data analysis Portal (UALCAN) proteomic datasets were analyzed to conduct pan-cancer analysis of IRP2. Pan-cancer analysis showed that IRP2 expression was elevated in lung cancer compared with normal tissues (Fig. 1A). IRP2 expression was higher in tumors than normal tissues in LUAD and lung squamous cell carcinoma (LUSC), suggesting a potential association with NSCLC progression (Fig. 1B and C). The Tumor Immune System Interaction Database (TISIDB) was used to evaluate the prognostic association of IRP2 in NSCLC. IRP2 expression was negatively correlated with overall survival in LUAD, whereas no significant association was observed in LUSC, indicating that IRP2 might serve as a poor prognostic factor particularly in LUAD (Fig. 1D and E).

We further examined the distribution of IRP2 within the TME. Through analysis of Tumor Immune Single-cell Hub 2 (TISCH2) and multiple Gene Expression Omnibus (GEO) datasets, IRP2 expression varied across cell types, with relatively high expression in monocytes/macrophages populations (Fig. S2A). Given that TAMs are correlated with tumor progression and prognosis, whereas the role of IRP2 in TAMs in NSCLC remains unclear^35^, we next assessed IRP2 expression in TAMs. NSCLC patients who underwent pulmonary resection at Nanjing Jinling Hospital were enrolled, and the expression levels of IRP2 in paired human tumors and paratumoral tissues were assessed by immunofluorescence. IRP2 was highly expressed in CD68^+^ macrophages and tumor cells in tumor tissues, and IRP2 expression in TAMs was 1.5-fold that in control macrophages (Fig. 1F, G and S2B). Collectively, these data suggest that elevated IRP2 expression is correlated with worse prognosis in NSCLC and TAMs represent an important IRP2-expressing cellular compartment within the TME.

### *Irp2* knockout in TAMs attenuates the progression of LLC tumors

To evaluate the role of IRP2 in TAMs, we generated *Irp2* myeloid-specific knockout mice,* Irp2^LysMCre^* mice, and established subcutaneous LLC tumor models in *Irp2^loxp/loxp^* mice and *Irp2^LysMCre^* mice. The knockout efficiency of IRP2 was validated by Western blot analysis of the BMDMs from *Irp2^loxp/loxp^* and* Irp2^LysMCre^* mice (Fig. S3). Compared with *Irp2^loxp/loxp^* mice, tumors in* Irp2^LysMCre^* mice exhibited markedly delayed growth, reduced tumor volumes, and an approximately 60% decrease in tumor weight at the experimental endpoint (Fig. 2A-C). These data indicate that myeloid *Irp2* deficiency suppresses LLC tumor progression *in vivo*.

To further characterize the biological changes resulting from *Irp2* deletion in TAMs, we assessed tumor cell proliferation and apoptosis in tumor tissues. IHC analysis showed that the density of Ki-67^+^ cells was reduced and Caspase-3 expression was significantly increased in tumors from *Irp2^LysMCre^* mice compared with* Irp2^loxp/loxp^* mice (Fig. 2D-F). Consistently, immunofluorescence analysis confirmed increased Caspase-3 expression in tumor cells of *Irp2^LysMCre^* tumor (Fig. 2G and H). Taken together, these findings demonstrate that *Irp2* deficiency in TAMs inhibits LLC tumor progression by reducing tumor cell proliferation and enhancing activation potential of apoptotic signaling.

### *Irp2* deficiency in macrophages promotes the M1 polarization of TAMs

We sought to explore the mechanisms by which* Irp2* deficiency in TAMs contributed to tumor cell death. First, we verified whether the portion of immune cells changed due to *Irp2* deficiency in TAMs. F4/80 IHC analysis and flow cytometric analysis consistently showed that the abundance of F4/80^+^ macrophage was not significantly changed by *Irp2* knockout (Fig. 3A-D). In addition, proportions of peripheral monocyte and granulocytes showed no significant difference between *Irp2^LysMCre^* mice and *Irp2^loxp/loxp^* mice, which implies that the inhibition of tumor growth was independent of change of myeloid cell abundance (Fig. S4A and B). We then assessed whether *Irp2* deficiency affects TAMs polarization by flow cytometry in subcutaneous tumors. The proportion of CD86^+^ macrophages was significantly increased from about 21.7% in *Irp2^loxp/loxp^* mice to 30.4% in *Irp2^LysMCre^* mice, whereas CD206^+^ macrophages showed no obvious differences (Fig. 3E-H). These results indicate that *Irp2* deficiency skews TAMs toward a more M1-like phenotype *in vivo*.

To further validate *in vitro*, BMDMs were stimulated with LLC-CM to generate TAMs. Flow cytometry analysis showed that *Irp2* deficiency increased the proportion of CD86^+^ TAMs from 25.1% in *Irp2^loxp/loxp^* TAMs to 43.3% in *Irp2^LysMCre^* TAMs, while the proportion of CD206^+^ TAMs in *Irp2^LysMCre^* TAMs displayed no significant difference compared with *Irp2^loxp/loxp^* TAMs (Fig. S4D-G). To explore mechanistic insight into this phenotypic reprogramming, transcriptome sequencing followed with KEGG enrichment analysis was performed. KEGG enrichment analysis showed that the TNF signaling pathway related to M1 polarization was significantly enriched in *Irp2^LysMCre^* TAMs (adjusted *P* < 0.05) (Fig. 3I). Consistently, the mRNA expression of *Il6*, *Tnf* and *Nos2* (M1 macrophage markers) was upregulated to 1.71-, 2.58-, and 1.44-fold respectively, and the mRNA expression of *Ym1* and *Arg1* (M2 macrophage markers) showed no significant difference in *Irp2^LysMCre^* TAMs compared with *Irp2^loxp/loxp^* TAMs (Fig. 3J). These data provide evidence that *Irp2* deficiency promotes M1 polarization of TAMs, potentially through modulation of the TNF signaling pathway.

### *Irp2*-deficient macrophages suppress tumor cell proliferation and migration while promoting apoptosis

Previous studies indicate that M1-like macrophages suppress tumor cell growth^36^, ^37^. Given that our results showed that *Irp2* deficiency reprogrammed TAMs toward a more M1 phenotype, we sought to explore whether this phenotypic shift was accompanied by functional changes affecting tumor cell behavior. BMDMs were co-cultured with LLC cells using Transwell inserts for subsequent experiments.

We first examined whether *Irp2*-deficient TAMs affected tumor cell migratory capacity. Scratch assays showed that LLC cells cultured with TAMs exhibited enhanced migration compared with LLC cells cultured in DMEM. Compared with LLC cells co-cultured with *Irp2^loxp/loxp^* TAMs, the migration rates of LLC cells co-cultured with *Irp2^LysMCre^* TAMs was reduced to approximately 55.8% and 78.4% at 24 h and 48 h, respectively. Thus, *Irp2* deficiency attenuated the pro-migratory effect of TAMs (Fig. 4A-C). Colony formation assay with crystal violet staining revealed that LLC cells co-cultured with *Irp2^LysMCre^* TAMs formed approximately 33.9% fewer colonies than those co-cultured with *Irp2^loxp/loxp^* TAMs (Fig. 4D and E). *Irp2*-deficient TAMs were less effective in maintaining the clonogenic growth of LLC cells. To further determine whether these changes were associated with altered tumor cell survival, we performed Annexin V-FITC/PI apoptosis assays. Compared with LLC cells cultured in DMEM, LLC cells co-cultured with *Irp2^loxp/loxp^* TAMs exhibited significantly a reduced apoptotic rate (8.07%), whereas those co-cultured with *Irp2^LysMCre^* TAMs exhibited a markedly higher apoptotic rate (24.57%) (Fig. 4F and G).

Thus, deletion of *Irp2* in TAMs not only abolished the anti-apoptotic effect of TAMs on tumor cells, but also shifted them toward a phenotype that favored tumor cell death. Taken together, these results demonstrate that *Irp2*-deficient TAMs exhibit reduced tumor-supportive activity and instead exert a direct suppressive effect on LLC cells by limiting migration and proliferative capacity while promoting apoptosis. These functional findings are consistent with the observed M1-like repolarization of *Irp2*-deficient TAMs and further support the conclusion that IRP2 contributes to the protumoral function of TAMs.

### *Irp2* deficiency in TAMs promotes T cell immune responses

Macrophages not only function in phagocytosing pathogenic microorganisms but also serve as important antigen-presenting cells highly expressing MHC-II and co-stimulatory molecules in TME. To assess the effect of IRP2 on the phagocytic capacity of TAMs, LLC-CM-induced TAMs were co-cultured with FluoSpheres™ polystyrene microspheres. The flow cytometry analysis showed that *Irp2^LysMCre^* TAMs exhibited enhanced phagocytosis compared with *Irp2^loxp/loxp^* TAMs (1.51-fold) (Fig. 5A and B). We next examined whether *Irp2* deficiency also affected the antigen-presenting phenotype of TAMs. RT-qPCR analysis showed that although *Cd74* expression was not significantly altered, the mRNA expression of *CD80* and *CD86* which provide co-stimulatory signals required for effective T-cell activation, was significantly upregulated in *Irp2^LysMCre^* TAMs, reaching 1.69-fold and 1.55-fold the levels observed in* Irp2^loxp/loxp^* TAMs, respectively (Fig. 5C). To further validate this observation *in vivo*, we assessed the antigen-presenting capacity of macrophages in subcutaneous tumor tissues. Flow cytometry analysis revealed increased proportion of MHC-II^+^ TAMs in tumors from* Irp2^LysMCre^* mice, corresponding to approximately 1.72-fold that observed in *Irp2^loxp/loxp^* tumors, suggesting enhanced antigen-presenting capacity of TAMs after *Irp2* knockout (Fig. 5D and E). Consistent with the *in vivo* findings, LLC-CM-induced *Irp2^LysMCre^* TAMs also displayed a higher proportion of MHC-II⁺ cells *in vitro* compared with *Irp2^loxp/loxp^* TAMs (Fig. 5F and G). These data suggest that *Irp2* deficiency promotes a more functionally active TAM phenotype characterized by improved antigen handling and greater T-cell-stimulatory potential.

In light of these findings, we next sought to explore whether the reprogramming of TAMs induced by *Irp2* deficiency was associated with altered T-cell responses. Flow cytometry showed that the proportions of CD4^+^ T cells and CD8^+^ T cells in tumors from *Irp2^LysMCre^* mice were 1.36 and 1.38 times those in* Irp2^loxp/loxp^* mice, respectively (Fig. 5H-J). In addition, spleen CD8^+^ T cells were increased (Fig. S5A-B), and the proportion of peripheral lymphocyte was also elevated in *Irp2^LysMCre^* mice compared with *Irp2^loxp/loxp^* mice (Fig. S4C). Moreover, CD8^+^ T cells in subcutaneous tumor from *Irp2^LysMCre^* mice exhibited increased expression of granzyme B (GZMB), indicating enhanced cytotoxic function (Fig. 5K and L). Taken together, these data support an immunosuppressive role of macrophage IRP2 in constraining T cell-mediated tumor surveillance.

### IRP2 regulates PD-L1 expression in TAMs

Given that the PD-1/PD-L1 axis mediates potent immunosuppressive signaling that impairs T cell functions, we subsequently investigated whether IRP2 contributes to the immunosuppressive phenotype of TAMs by regulating PD-L1 expression. Immunofluorescence staining of subcutaneous tumor tissues showed that PD-L1 expression in TAMs from *Irp2^LysMCre^* mice was decreased by approximately 82% compared with that in TAMs from* Irp2^loxp/loxp^* mice (Fig. 6A and B). To further validate this result, flow cytometric analysis revealed a significantly decreased proportion of PD-L1^+^ TAMs in subcutaneous tumors from about 33.4% in *Irp2^loxp/loxp^* mice to 15.6% in *Irp2^LysMCre^* mice (Fig. 6C and D). Additionally, LLC-CM-induced TAMs from *Irp2^LysMCre^* mice exhibited a decreased proportion of PD-L1^+^ TAMs, corresponding to 0.79-fold that observed in TAMs from *Irp2^loxp/loxp^* mice (Fig. S6A and B). These findings suggest that IRP2 in TAMs might modulate T-cell activation and function by regulating membrane PD-L1 expression.

To investigate how *Irp2* deficiency affected PD-L1 abundance, we compared BMDMs maintained in DMEM with LLC-CM-induced TAMs. Under basal DMEM conditions, *Irp2* deficiency increased PD-L1 expression in BMDMs by approximately 47% compared with *Irp2^loxp/loxp^* controls. However, this pattern was reversed under LLC-CM stimulation. LLC-CM markedly increased PD-L1 expression in *Irp2^loxp/loxp^* TAMs by approximately 36% compared with *Irp2^loxp/loxp^* BMDMs, whereas PD-L1 levels in* Irp2^LysMCre^* TAMs was approximately 35% lower than that in *Irp2^loxp/loxp^* TAMs. Notably, *Irp2^LysMCre^* TAMs maintained significantly reduced PD-L1 expression compared with *Irp2^LysMCre^* BMDMs, with an approximately 40% reduction. (Fig. 6E and F). This differential response under basal and tumor-conditioned conditions highlights the context-dependent regulation of PD-L1 by IRP2.

PD-L1 expression is coordinately regulated at multiple levels, including transcriptional regulation and ubiquitin-mediated proteasomal degradation. To evaluate transcriptional regulation, we quantified *Cd274*, the gene encoding PD-L1 via RT-qPCR. *Irp2* knockout significantly suppressed *Cd274* transcription under basal DMEM conditions, whereas no significant difference of *Cd274* expression was observed under LLC-CM conditions (Fig. 6G). Although transcriptional changes were context-dependent, the reduced PD-L1 expression in *Irp2*-deficient TAMs cannot be fully explained by transcriptional regulation alone. We therefore examined whether IRP2 disruption was associated with PD-L1 protein degradation.

Notably, *Irp2^LysMCre^* TAMs exhibited increased LAMP1 expression compared with *Irp2^loxp/loxp^* TAMs (1.17-fold), and Western blot analysis showed a modest increase in CTSB levels, indicating enhanced lysosomal activity (Fig. 6H-J). Consistent with these findings, additional Western blot analysis confirmed that PD-L1 expression was reduced and LAMP1 expression was increased in *Irp2^LysMCre^* TAMs compared with *Irp2^loxp/loxp^* TAMs (Fig. S6C). To further explore whether elevated protein degradation contributed to the reduction of PD-L1 in *Irp2*-deficient TAMs, we treated TAMs with the lysosomal inhibitors CQ or the proteasome inhibitor MG132. Both CQ and MG132 partially alleviated the downregulation of PD-L1 by IRP2 disruption in *Irp2^LysMCre^* TAMs, as demonstrated by immunofluorescence staining and Western blot analysis (Fig. 6K and L; Fig. S6D). These results suggest that IRP2 may regulate PD-L1 through lysosome- and proteasome-dependent degradation in TAMs.

To further evaluate whether lysosome-associated regulation contributes to the antitumor effects mediated by *Irp2* deficiency in TAMs, we performed pharmacological intervention using CQ in the LLC subcutaneous tumor model. *Irp2^loxp/loxp^* and *Irp2^LysMCre^* mice bearing tumor were treated with CQ by intraperitoneal injection. CQ treatment markedly inhibited tumor growth in *Irp2^loxp/loxp^* mice, whereas this tumor-suppressive effect was diminished in *Irp2^LysMCre^* mice (Fig. S7A-C). IHC analysis further showed that tumors from *Irp2^LysMCre^* mice exhibited higher infiltration of CD4^+^ and CD8^+^ T cells than those from *Irp2^loxp/loxp^* mice, consistent with our previous results. After CQ treatment, Caspase-3 and infiltration of Ki-67^+^ cells showed no obvious changes in *Irp2^loxp/loxp^* mice, whereas macrophage and CD4^+^, CD8^+^ T cell infiltration were significantly increased (3.07-, 3.84-, and 2.51-fold, respectively). In contrast, in tumors of *Irp2^LysMCre^* mice, CQ treatment further increased Caspase-3 expression (1.52- fold), while proportion of Ki-67^+^ cells and macrophage showed non-significant increases. CD4^+^ T cell infiltration was significantly decreased (0.51-fold) and CD8^+^ T cell infiltration showed no significant difference. Although CQ may affect multiple cellular compartments within TME, the differential responses to CQ suggest that lysosome-associated pathways participate in the antitumor regulation mediated by IRP2 of TAMs (Fig. S7D-I).

### *Irp2* deficiency promotes PD-L1 degradation in TAMs via the SphK1-lysosome axis

To explore the downstream pathways by which *Irp2* deficiency promotes PD-L1 degradation in TAMs, RNA sequencing (RNA-seq) was performed to compare *Irp2^loxp/loxp^* and *Irp2^LysMCre^* TAMs induced by LLC-CM. Differential expression analysis identified 265 differentially expressed genes (DEGs) (Fig. 7A). GO enrichment analysis showed that these DEGs were significantly enriched in pathways related to the G protein-coupled receptor signaling pathways (Fig. S8A). Among the DEGs, we focused on *Sphk1* which encodes a rate-limiting enzyme in sphingolipid metabolism with a reported role in maintaining lysosomal membrane homeostasis and function^38^, ^39^. RT-qPCR analysis further confirmed that *Sphk1* mRNA expression in *Irp2^LysMCre^* TAMs was significantly upregulated compared with *Irp2^loxp/loxp^* TAMs (2.16-fold) (Fig. S8B). Consistent with these transcriptional findings, the expression of SphK1 in TAMs of tumors from *Irp2^LysMCre^* mice was increased (3.23-fold) (Fig. 7B and C).

To examine whether SphK1 is involved in IRP2-associated alterations in lysosomal function, TAMs were treated with selective SphK1 inhibitor PF-543. *Irp2^LysMCre^* TAMs treated with PF-543 showed partially restored CTSB levels and significantly reduced LAMP1 abundance compared with *Irp2^LysMCre^* TAMs, indicating that SphK1 inhibition attenuated the lysosome-associated phenotype caused by *Irp2* deficiency (Fig. 7D-F). These findings suggest that SphK1 contributes to the regulation of lysosomal function downstream of IRP2. Given the impact of PF-543 on lysosomal activity, we further investigated whether SphK1-mediated lysosomal regulation influences PD-L1 expression. The *Irp2^LysMCre^* TAMs treated with PF-543 significantly increased PD-L1 expression compared with untreated controls (2.84-fold) (Fig. 7G and H). Consistent with the immunofluorescence results, western blot analysis further confirmed the corresponding changes in LAMP1 and PD-L1 expression (Fig. 7I). Together, these data suggest that SphK1 may contribute to the lysosome-associated regulation of PD-L1 expression.

To further evaluate the *in vivo* relevance of SphK1 signaling in IRP2-mediated antitumor immunity, LLC tumor-bearing mice were treated intraperitoneally with PF-543. PF-543 treatment markedly suppressed subcutaneous tumor growth in *Irp2^loxp/loxp^* mice, whereas no significant change was observed in* Irp2^LysMCre^* mice after PF-543 intervention (Fig. S7A-C). This diminished response to PF-543 suggests that SphK1-associated sphingolipid metabolic pathways may partially overlap with the antitumor mechanisms triggered by *Irp2* deletion in TAMs. IHC analysis further revealed distinct effects of PF-543 on apoptosis, proliferation, and immune-cell infiltration. In *Irp2^loxp/loxp^* tumors, PF-543 treatment increased Caspase-3 expression (1.76-fold), without significantly altering the proportion of Ki-67^+^ cells. Meanwhile, macrophage and CD4^+^ T-cell infiltration showed a slight but non-significant increase, whereas CD8^+^ T-cell infiltration was significantly elevated after PF-543 treatment (4.02-fold). By contrast, the response to PF-543 was markedly different in *Irp2^LysMCre^* tumors. PF-543 treatment significantly decreased Caspase-3 expression (0.31-fold), while infiltration of macrophages and Ki-67^+^ cells showed no significant change, and abundance of both CD4^+^ and CD8^+^ T cells were significantly reduced in *Irp2^LysMCre^* tumors (0.16- and 0.39-fold, respectively) (Fig. S7D-I). These findings suggest that the antitumor and immunomodulatory effects of PF-543 are influenced by TAM *Irp2*, further supporting the involvement of the IRP2-SphK1 axis in regulating the tumor immune microenvironment *in vivo*.

To address whether SphK1-mediated regulation of PD-L1 is restricted to TAMs or also occurs in other cell types within the TME, we additionally examined the effect of SphK1 inhibition in LLC, Jurkat, RAW 264.7, and A549 cells. After treatment with PF-543 for 4 h, PD-L1 expression was altered in LLC, A549, and RAW 264.7 cells. PF-543 treatment reduced LAMP1 protein levels in LLC and RAW 264.7 cells, but did not markedly affect LAMP1 expression in Jurkat or A549 cells (Fig. S8C-F). These results suggest that SphK1 inhibition is associated with PD-L1 expression beyond TAMs, while its effect on lysosome-associated protein expression appears to be cell type-dependent.

### Sphingolipid metabolic remodeling contributes to lysosome-associated PD-L1 degradation in* Irp2* deficient TAMs

To clarify the metabolic mechanism underlying IRP2-mediated regulation of the SphK1-PD-L1 axis, we further performed lipidomic profiling of *Irp2^loxp/loxp^* and *Irp2^LysMCre^* TAMs induced by LLC-CM and detected sphingolipid-related metabolites. The results showed that *Irp2* deficiency markedly altered the sphingolipid metabolic profile of TAMs, as reflected by changes in multiple sphingomyelin- and ceramide-related lipid species (Fig. 8A). In *Irp2^loxp/loxp^* TAMs, PMA was used to induce SphK1 expression and was accompanied by reduced PD-L1 expression (Fig. S9A-B). SphK1 is a key enzyme responsible for the generation of S1P, and S1P has been reported to participate in lysosomal homeostasis and autophagy-related degradation. Therefore, TAMs were further treated with S1P to functionally determine whether S1P could regulate lysosomal function and PD-L1 expression. Immunofluorescence analysis showed that S1P treatment increased LAMP1 abundance in *Irp2^loxp/loxp^* TAMs and *Irp2^LysMCre^* TAMs relative to the corresponding untreated controls. CTSB expression was not significantly altered after S1P treatment in either *Irp2^loxp/loxp^* or *Irp2^LysMCre^* TAMs (Fig. 8B-D). Accordingly, S1P reduced PD-L1 expression of *Irp2^loxp/loxp^* and *Irp2^LysMCre^* TAMs (0.81- and 0.72-fold, respectively) (Fig. 8E-F). These results support the possibility that S1P may enhance lysosome-associated activity and contribute to the downregulation of PD-L1 in TAMs. Together with the aforementioned SphK1 inhibition experiments, these data further support an association between *Irp2* deficiency and enhanced PD-L1 degradation potentially involving sphingolipid metabolic remodeling.

Considering that PD-L1 expression on TAMs may contribute to ICI resistance, we further investigated the response to anti-PD-1 treatment in *Irp2^loxp/loxp^* and* Irp2^LysMCre^* tumor-bearing mice. After LLC implantation, mice received intraperitoneal injections of PBS or anti-PD-1 monoclonal antibody respectively. Compared with the corresponding control groups, anti-PD-1 treatment further inhibited tumor progression, reducing subcutaneous tumor weight in *Irp2^loxp/loxp^* and *Irp2^LysMCre^* mice (Fig. 8G-I), suggesting a more pronounced antitumor effect of PD-1 blockade in *Irp2^LysMCre^* mice.

## Discussion

PD-1/PD-L1 blockade has become an important therapeutic strategy for advanced NSCLC. However, resistance to PD-1/PD-L1 blockade continues to limit its long-term efficacy. Beyond tumor-intrinsic mechanisms, TAM-mediated immunosuppression, particularly through TAM-derived PD-L1, can impair T-cell activation and shape the therapeutic response to ICIs. Our findings identify the important role of IRP2 in shaping the immunosuppressive state of TAMs and adaptive antitumor immunity in NSCLC. IRP2 may constrain T-cell-mediated antitumor responses, potentially in part through alterations in the antigen-presenting capacity of TAMs, consistent with the emerging concept that reprogramming immunosuppressive TAMs may alleviate microenvironment-driven resistance and enhance antitumor immunity^12^, ^40^-^42^. Mechanistically, our data support the involvement of the IRP2-SphK1 axis in regulating PD-L1 expression through lysosome-dependent degradation in TAMs. IRP2 deficiency is associated with SphK1 upregulation, enhanced lysosomal activity, and accelerated PD-L1 degradation in TAMs. These results provide a new molecular framework for understanding how TAMs promote tumor progression, and suggest that IRP2 in TAMs may represent a promising therapeutic target for improving the therapeutic response to ICIs in NSCLC (Fig. 9).

Iron availability is essential for sustaining the proliferation, growth, and survival of tumor cells within the TME^43^. As a central regulator of cellular iron homeostasis, IRP2 coordinates iron uptake, storage, and export. Previous studies have reported that elevated IRP2 expression in tumor cells is associated with tumor progression and poor prognosis in NSCLC^27^, ^44^. Although TAMs constitute a major iron-responsive immune population within the TME, the functional significance of IRP2 in TAMs remains incompletely understood^45^, ^46^. Our findings extend the biological relevance of IRP2 beyond iron homeostasis by identifying it as an important regulator of TAM immune function.

TAMs are functionally plastic, with M2-like states generally supporting tumor progression and immune suppression, whereas M1-like states can promote antitumor immunity through phagocytosis, antigen presentation, and the activation of cytotoxic lymphocytes^47^-^49^. Previous work suggested that IRP2 in macrophages is essential for immune responses under inflammatory conditions^29^. Our data provide further evidence that *Irp2* deficiency reprograms TAMs towards an M1-like phenotype, characterized by elevated CD86 expression and enhanced phagocytic capacity. Although* Irp2* deficiency is generally expected to reduce transferrin receptor 1 (TfR1) expression and increase ferritin expression, thereby limiting intracellular iron availability, these changes are not necessarily inconsistent with an M1-like macrophage phenotype. Previous studies have shown *Irp2* deficiency can cause tissue or cellular iron accumulation while simultaneously producing functional iron deficiency because iron is sequestered in ferritin^28^, ^29^. Inflammatory macrophage activation is accompanied by reduced IRP activity, decreased TfR1 expression, and increased ferritin expression^50^, ^51^. Moreover, iron supplementation did not restore lysosome-related proteins in *Irp2*-deficient macrophages, suggesting that IRP2-dependent macrophage phenotypes cannot be explained solely by iron availability^29^. Therefore, the M1 reprogramming of *Irp2*-deficient TAMs was defined based on their phenotypic and functional characteristics rather than by cellular iron status. Concurrently, these *Irp2*-deficient TAMs exert direct antitumor effects by inhibiting cancer cell proliferation and migration while promoting apoptosis. We found that *Irp2* deficiency enhanced antigen-presenting capacity of TAMs. Given the importance of antigen uptake and presentation in priming tumor-specific T cells and eliciting antitumor immune responses^52^, this functional reprogramming may partly explain the increased infiltration of CD8⁺ T cells observed in *Irp2^LysMCre^* mice.

Beyond antigen presentation, *Irp2*-deficient TAMs may influence T-cell responses through additional mechanisms. PD-L1 expressed by TAMs has emerged as an important contributor to the dysfunction of tumor-infiltrating effector cells in NSCLC^53^-^55^. Our study describes a novel pathway through which IRP2 contributes to the immunosuppressive phenotype of TAMs. Our data suggest that IRP2 helps maintain PD-L1 expression on TAMs, at least partly by limiting lysosome-associated degradation. The partial restoration of PD-L1 by MG132 further suggests that proteasome-dependent turnover may also contribute, although the present study mainly supports a lysosome-associated mechanism.

More broadly, IRP2-dependent TAM reprogramming may influence the immunological consequences within the TME in addition to its potential macrophage-intrinsic effects on PD-L1 stability. Thus, alterations of TAM IRP2 may propagate beyond macrophages and contribute to broader changes in the tumor immune landscape. Given that other immune cell populations were not comprehensively characterized in the present study, the cellular basis of this immune remodeling remains to be determined.

To explore the molecular link between IRP2 and lysosome-dependent PD-L1 degradation, we further focused on SphK1, whose metabolic product S1P has been reported to be involved in TRAF2-BECN1-dependent autophagy and lysosomal regulation^38^. We analyzed the annotated 5′ and 3′ UTRs of all mouse *Sphk1* RefSeq transcripts using SIREs 3.0. No candidate IRE was predicted in any of the analyzed transcripts. Thus, the sequence analysis does not support a canonical direct IRP2-IRE mechanism and suggests that the increase of *Sphk1* mRNA following *Irp2* deficiency may instead arise through an indirect regulatory mechanism. Our findings suggest that SphK1-related signaling contributes to the IRP2-dependent regulation of lysosomal activity and PD-L1 stability in TAMs. The effects of SphK1 inhibition and S1P supplementation, together with alterations in ceramide- and sphingomyelin-related lipid species, further suggest that *Irp2* deficiency is associated with broader remodeling of sphingolipid metabolism. Collectively, these findings support the important role of the IRP2-SphK1-lysosome pathway in regulating PD-L1 degradation and immunosuppression of TAMs.

From a translational perspective, targeting the IRP2-SphK1-PD-L1 axis within TAMs may offer a complementary strategy for improving immunotherapy in NSCLC. Rather than acting primarily through direct tumor-cell cytotoxicity, IRP2 modulation would be expected to remodel the immunosuppressive TME. A multimodal strategy that combines extracellular checkpoint blockade with approaches that reduce intracellular PD-L1 accumulation may be more effective than checkpoint blockade alone^56^. In this context, combining TAM-directed IRP2 targeting with immune checkpoint blockade may simultaneously limit PD-L1 accumulation in TAMs and disrupt PD-1/PD-L1-mediated T-cell suppression. IRP2 modulation may also complement other therapeutic approaches that promote antigen release, immune infiltration, or metabolic reprogramming. However, these combination strategies require further validation in clinically relevant NSCLC models and human-derived TAM systems. Although the development of IRP2-targeted therapies remains constrained by the lack of selective pharmacological agents, recent evidence suggests that the small-molecule inhibitor KS20226 can reprogram iron metabolism and suppress tumor growth in a colorectal cancer model^57^. Targeting SphK1-related signaling may offer an alternative therapeutic intervention, although its translational relevance requires further validation. Given the essential role of iron metabolism in normal tissues, the clinical application of IRP2-targeted therapies will likely require TAM-selective delivery strategies to maximize intratumoral activity while minimizing systemic disruption of iron metabolism.

Several limitations should be acknowledged in our study. Our findings are primarily based on the LLC tumor model, which does not fully recapitulate the heterogeneity and complexity of human NSCLC. Therefore, further validation in human NSCLC specimens or clinically relevant human-derived models will be necessary. Furthermore, the present study mainly focuses on short-term tumor control rather than durable survival benefit. Future studies using long-term survival monitoring, postoperative recurrence models, and metastatic models will be needed to determine whether targeting IRP2 in TAMs can improve overall survival and long-term tumor control. Additionally, we did not perform comprehensive profiling of all major immune populations within the TME. Therefore, future studies incorporating broader immune profiling will be necessary to define the full impact of IRP2-dependent TAM reprogramming on the overall immune landscape of NSCLC.

Overall, our findings illustrate that IRP2 may function as a potential therapeutic target in NSCLC and a potential biomarker requiring further clinical validation.

## Conclusion

Together, our results identify IRP2 as an important regulator of TAM-mediated immunosuppression and support further evaluation of IRP2-SphK1-PD-L1 signaling as a promising TAM-directed therapeutic target in NSCLC, which might potentially benefit patients who exhibit resistance to ICI therapy.

## Supplementary Material

Supplementary figures and tables.

## Figures and Tables

**Figure 1:**
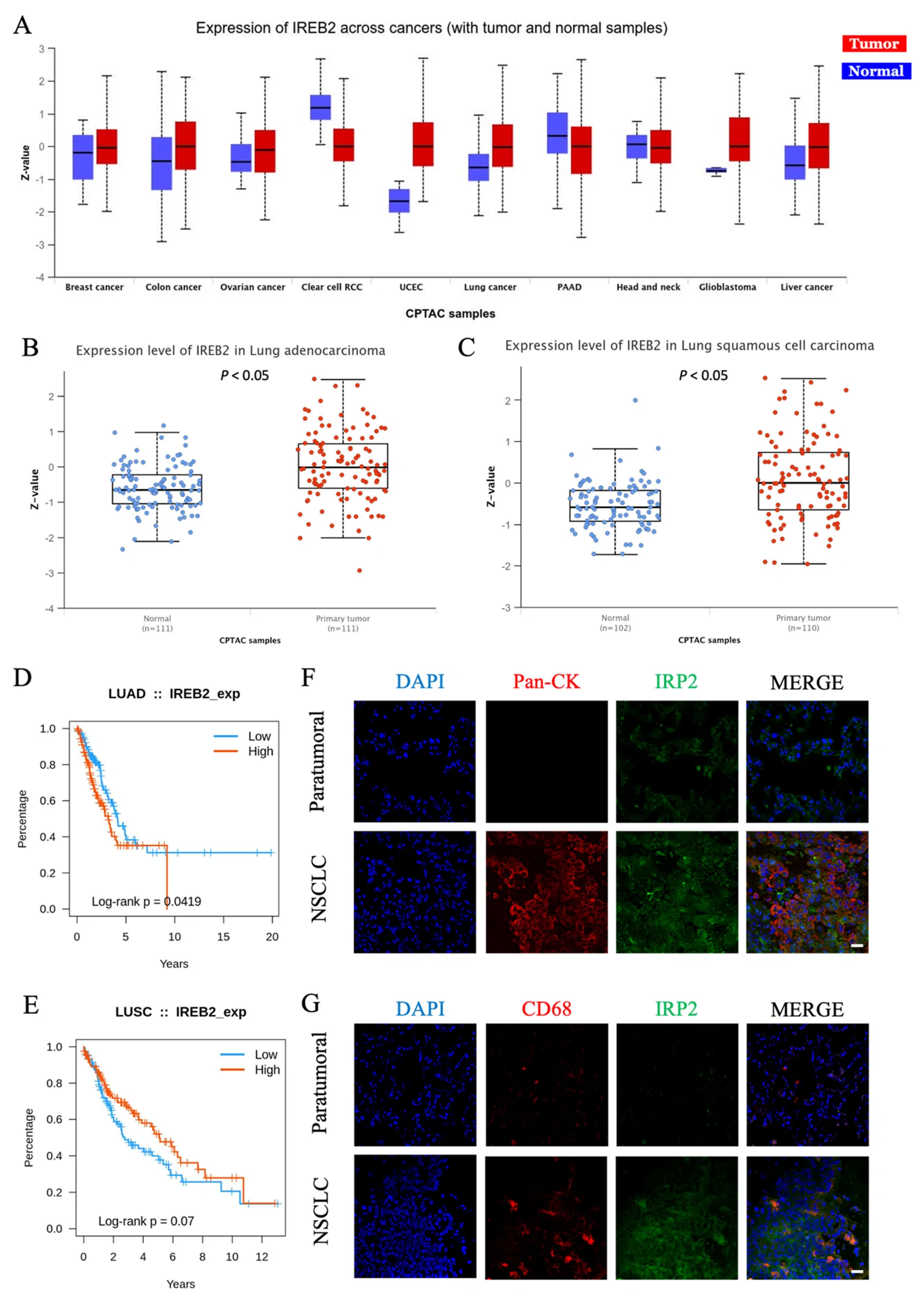
** IRP2's high expression is associated with poor prognosis in NSCLC patients.** (A) Expression levels of IRP2 in various cancer types, analyzed using the UALCAN database. (B-C) IRP2 expression in LUAD (B) and LUSC (C) samples respectively, analyzed via the UALCAN database. (D-E) Correlation between IRP2 expression levels and patient overall survival in LUAD (D) and LUSC (E) respectively, analyzed using the TISIDB database. (F-G) Representative immunofluorescence staining of IRP2 in tumor cells (F) and macrophages (G) in NSCLC tumor tissues and adjacent non-tumor tissues. Scale bar = 30 μm. *P* values were determined by two-tailed Student's t-test. Survival analyses were evaluated with the log-rank test. Statistical significance was defined as *P* < 0.05.

**Figure 2:**
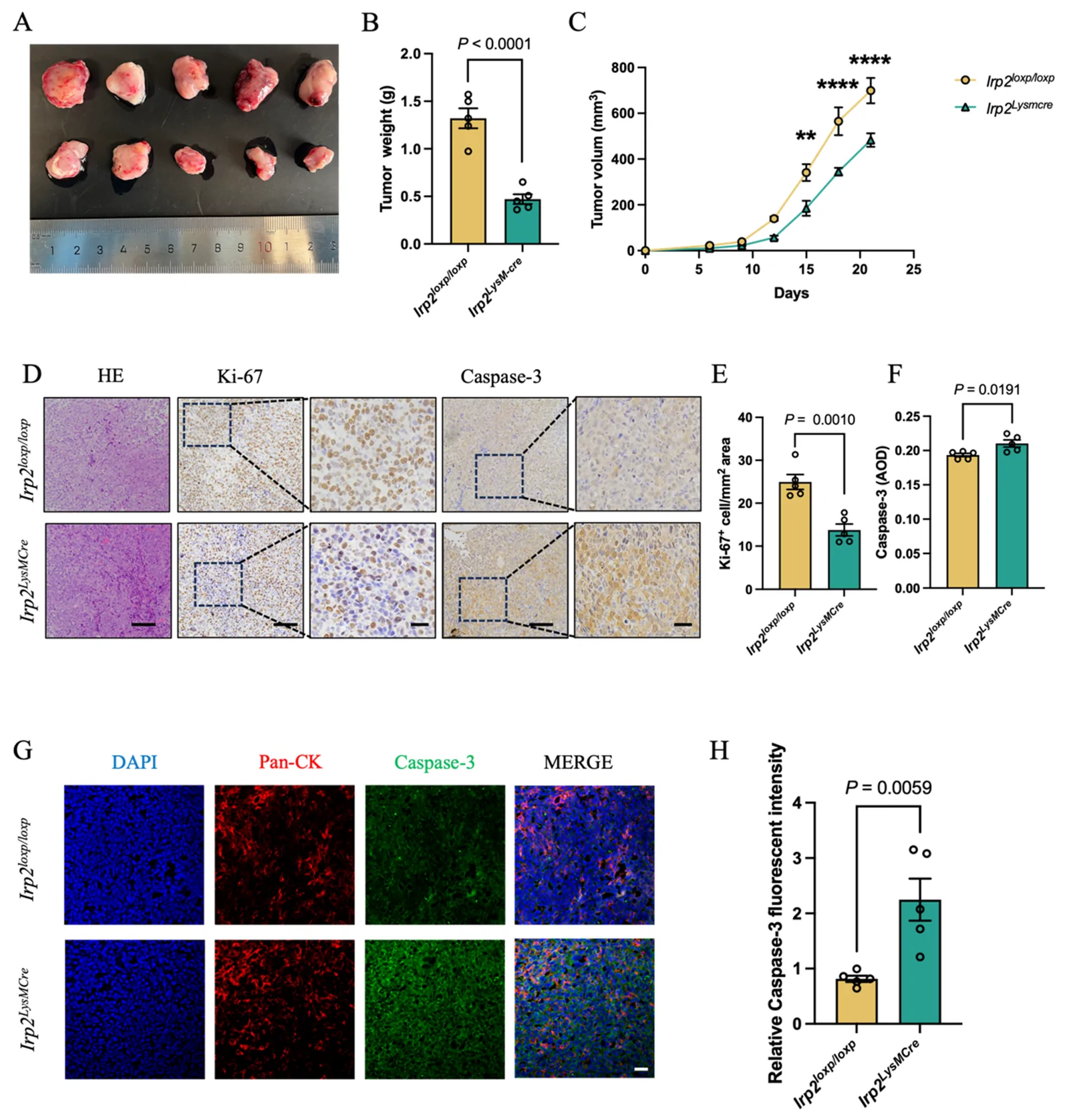
***Irp2* deficiency in macrophages suppresses LLC tumor growth.** (A) Representative images of subcutaneous tumor tissue from *Irp2^loxp/loxp^* and *Irp2^LysMCre^* mice. (B) Quantification of subcutaneous tumor weights. (C) Tumor growth curves showing tumor volume over time. (D) Representative images of H&E staining and IHC staining of Ki-67 and Caspase-3 expression in *Irp2^loxp/loxp^* and *Irp2^LysMCre^* tumor tissues. (E-F) Quantitative analysis of Ki-67^+^ cells (E; Hedges' g = -2.901) and Caspase-3 expression (F; Hedges' g = 1.672) in *Irp2^loxp/loxp^* and *Irp2^LysMCre^* tumor tissues. Scale bar = 50 μm. Scale bars in enlarged images = 15 μm. (G-H) Representative immunofluorescence staining (G) and quantitative analysis (H; Hedges' g = 2.126) of Caspase-3 expression in Pan-CK^+^ cells from *Irp2^loxp/loxp^* and *Irp2^LysMCre^* tumor tissues. Scale bar = 30 μm. Data are represented as mean ± SEM. *P* values were determined by two-tailed Student's t-test respectively. Statistical significance was defined as *P* < 0.05. Effect size was calculated as Hedges' g. n = 5.

**Figure 3:**
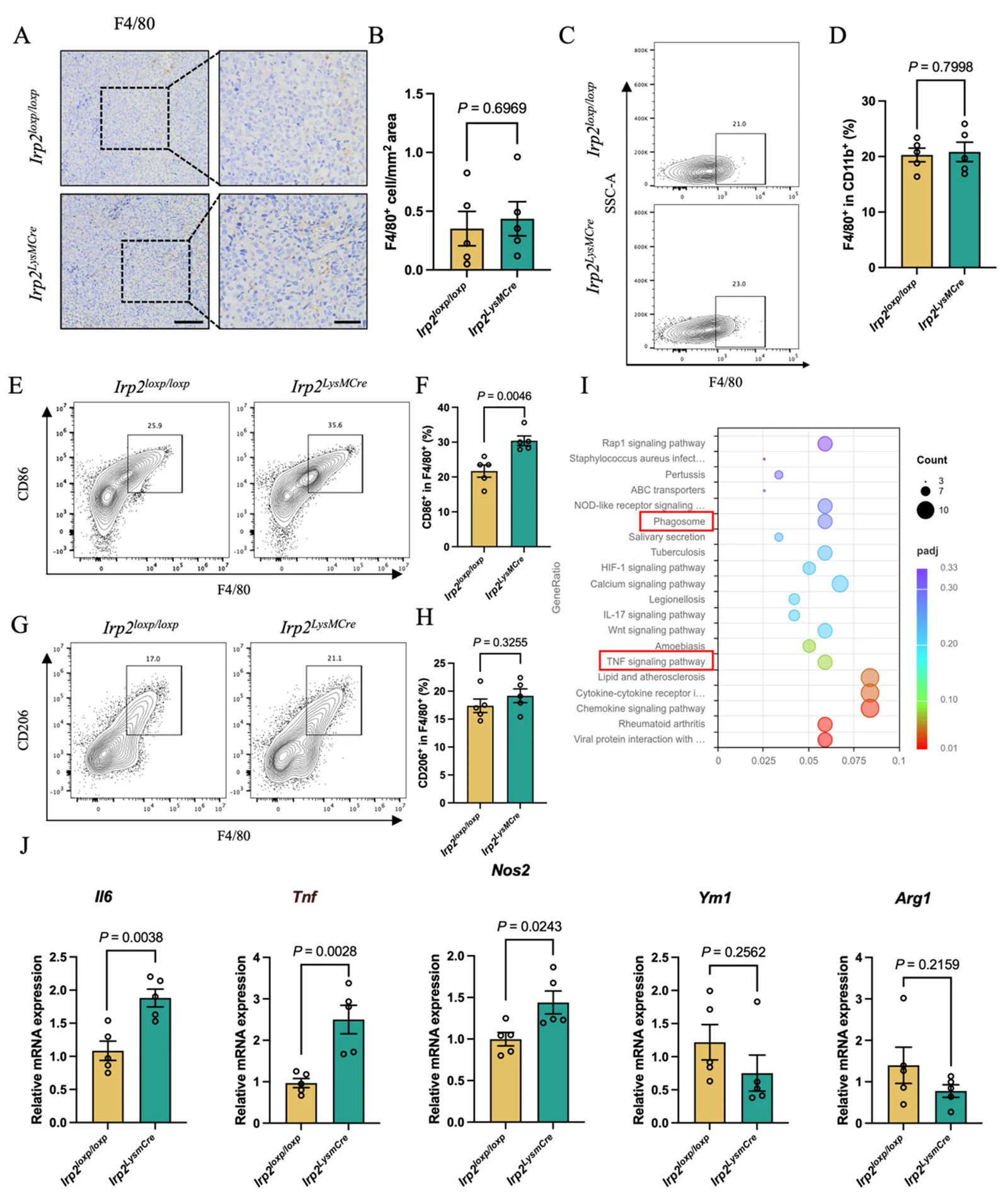
***Irp2* deficiency in macrophages promotes the M1 polarization of TAMs.** (A) Representative IHC staining of F4/80 in subcutaneous tumor tissues from *Irp2^loxp/loxp^* and *Irp2^LysMCre^* mice, with locally magnified images. Scale bar = 50 μm. Scale bars in enlarged images = 20 μm. (B) Quantitative analysis of the density of macrophages in subcutaneous tumor tissues. n = 5. (C-D) Representative flow cytometry plots (C) and quantitative analysis (D) of the infiltration ratio of macrophages in CD11b^+^ cells in subcutaneous tumor tissues from *Irp2^loxp/loxp^* and *Irp2^LysMCre^* mice. n = 5. (E-F) Representative flow cytometry plots (E) and quantitative analysis (F) of the proportion of CD86⁺ cells among macrophages in subcutaneous tumor tissues (Hedges' g = 2.226). n = 5. (G-H) Representative flow cytometry plots (G) and quantitative analysis (H) of the proportion of CD206⁺ cells among F4/80⁺ macrophages in subcutaneous tumor tissues. n = 5. (I) KEGG pathway enrichment analysis of differentially expressed genes in *Irp2^loxp/loxp^* and *Irp2^LysMCre^* TAMs after 48 h of induction with LLC-CM. (J) RT-qPCR detection of the mRNA expression levels of *Il6* (Hedges' g = 2.302), *Tnf* (Hedges' g = 2.429), *Nos2* (Hedges' g = 1.582), *Ym1*, and *Arg1* in *Irp2^loxp/loxp^* and *Irp2^LysMCre^* TAMs after 48 h of induction with LLC-CM. n = 5. Data are represented as mean ± SEM. *P* values were determined by two-tailed Student's t-test. Statistical significance was defined as *P* < 0.05. Effect size was calculated as Hedges' g.

**Figure 4:**
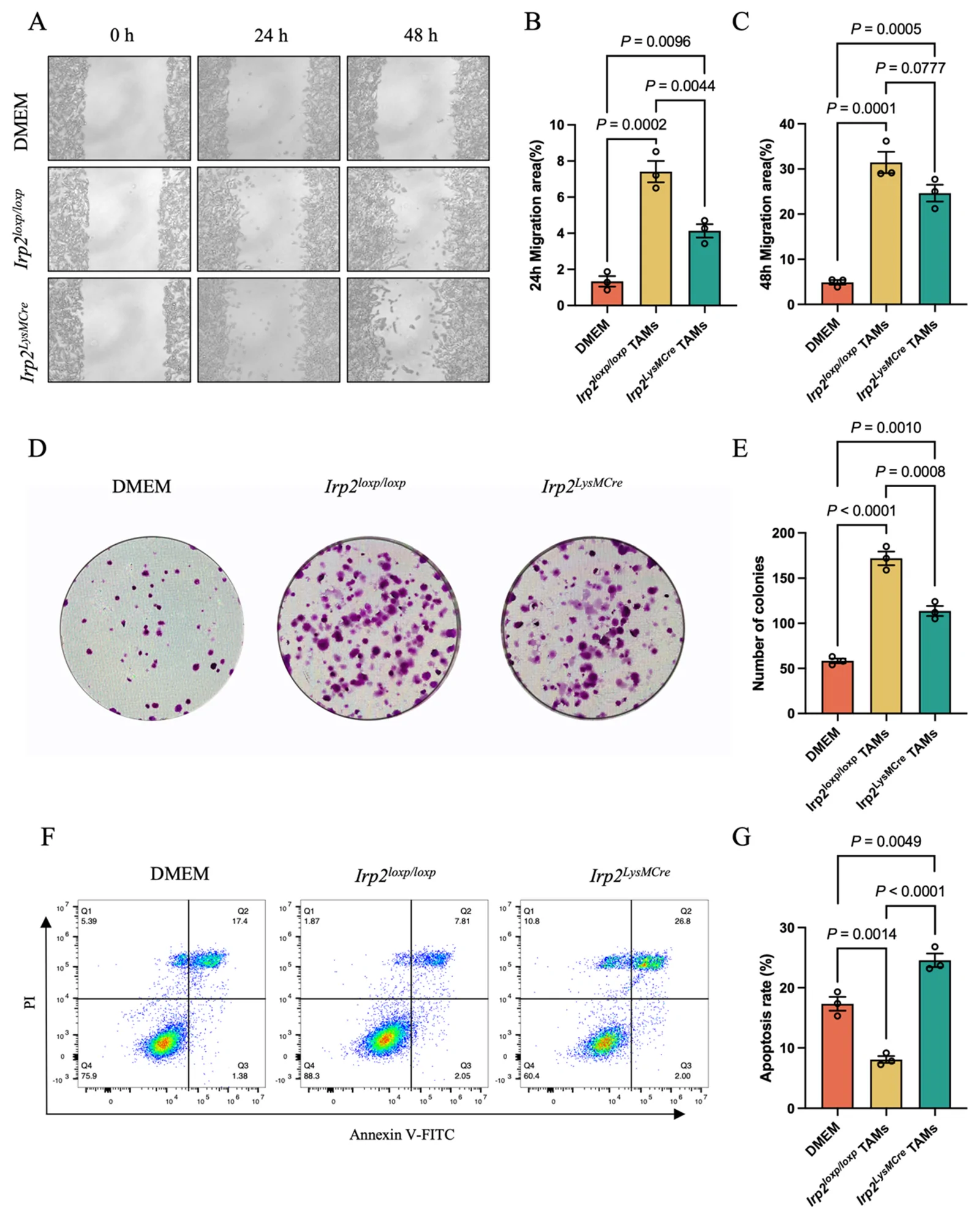
** Effects of *Irp2*-deficient macrophages on LLC cells.** (A-C) Representative images of scratch assay (A) and quantitative analysis of LLC cell migration rate at 24 h (B) and 48 h (C) under different culture conditions: DMEM as the blank control group, Transwell co-culture with *Irp2^loxp/loxp^* TAMs or *Irp2^LysMCre^* TAMs as the treated group. n = 3. (D-E) Representative images of colony formation assay (D) and quantitative analysis of colony formation (E) of LLC cells under different culture conditions: DMEM medium, Transwell co-culture with *Irp2^loxp/loxp^* TAMs or *Irp2^LysMCre^* TAMs. n = 3. (F-G) Representative flow cytometry plots (F) and quantitative analysis of late apoptosis (G) of LLC cells after treated with DMEM medium, CM from *Irp2^loxp/loxp^* TAMs or *Irp2^LysMCre^* TAMs. n = 3. Data are represented as mean ± SEM. *P* values were determined by one-way analysis of variance (ANOVA). Statistical significance was defined as *P* < 0.05.

**Figure 5:**
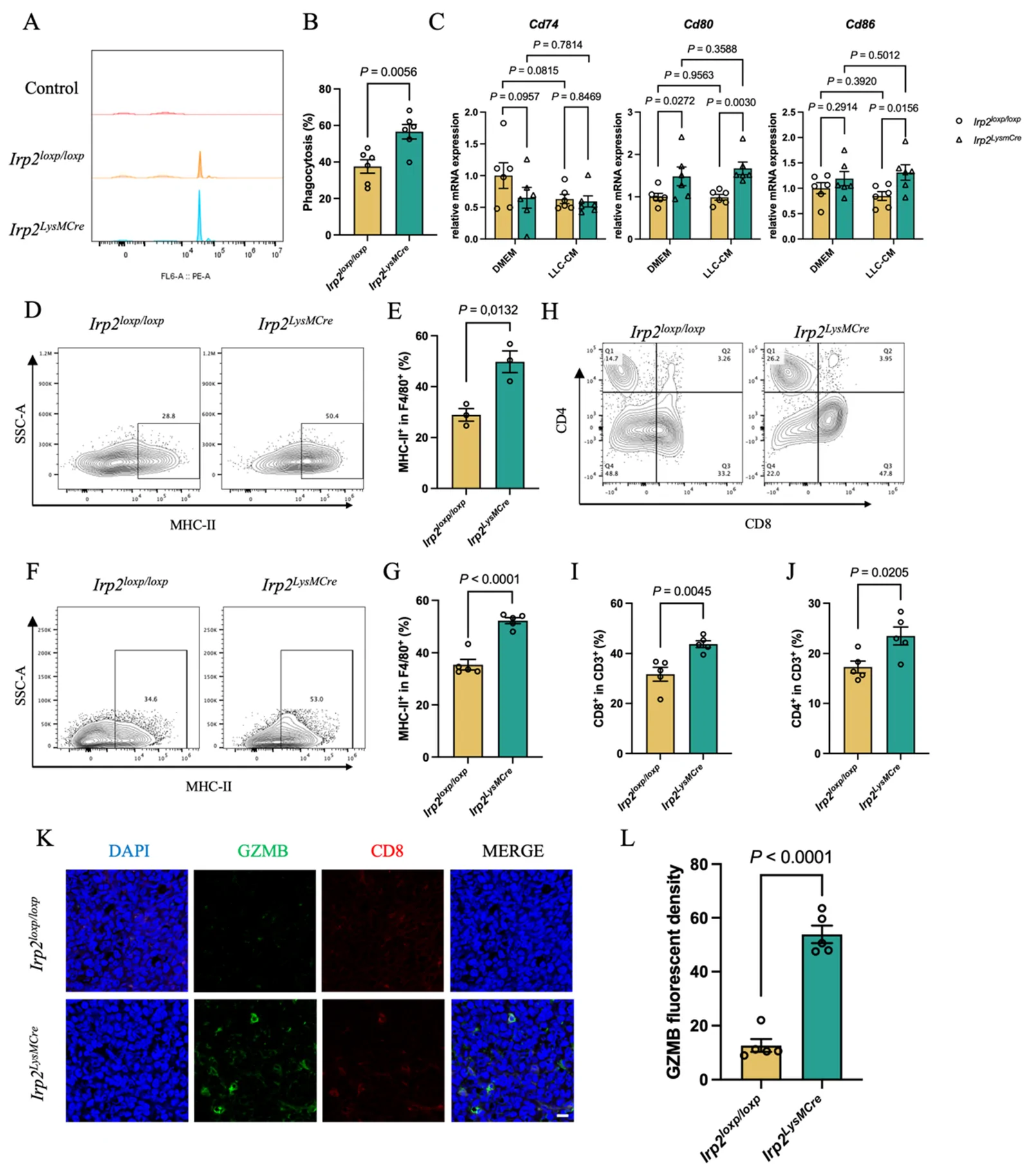
** IRP2 modulates TAM phagocytic capacity, antigen-presenting function, and antitumor T cell infiltration in subcutaneous tumors.** (A-B) Representative flow cytometry plots (A) and quantitative analysis (B) of the phagocytic uptake of FluoSpheres™ polystyrene microspheres by LLC-CM-induced *Irp2^loxp/loxp^* and *Irp2^LysMCre^* TAMs (Hedges' g = 1.874). n = 6. (C) RT-qPCR analysis of the mRNA expression levels of *Cd74*, *Cd80*, and *Cd86* in *Irp2^loxp/loxp^* BMDMs, *Irp2^LysMCre^* BMDMs, *Irp2^loxp/loxp^* TAMs, and *Irp2^LysMCre^* TAMs. n = 6. (D-E) Representative flow cytometry plots (D) and quantitative analysis (E) of the proportion of MHC-II⁺ TAMs in subcutaneous tumor tissues from *Irp2^loxp/loxp^* and *Irp2^LysMCre^* mice (Hedges' g = 2.775). n = 3. (F-G) Representative flow cytometry plots (F) and quantitative analysis (G) of the proportion of MHC-II⁺ TAMs in LLC-CM-induced *Irp2^loxp/loxp^* and *Irp2^LysMCre^* TAMs. n = 5. (H-J) Representative flow cytometry plots (H) and quantitative analysis of the infiltration proportions of CD8⁺ T cells (I; Hedges' g = 2.235) and CD4⁺ T cells (J; Hedges' g = 1.644) in subcutaneous tumor tissues from *Irp2^loxp/loxp^* and *Irp2^LysMCre^* mice. n = 5. (K-L) Representative immunofluorescence staining (K) and quantitative analysis (L) of granzyme B (GZMB) expression in CD8⁺ T cells in *Irp2^loxp/loxp^* and *Irp2^LysMCre^* mouse tumor tissues. Scale bar = 15 μm. n = 5. Data are represented as mean ± SEM. *P* values were determined by two-tailed Student's t-test or two-way ANOVA. Statistical significance was defined as *P* < 0.05. Effect size was calculated as Hedges' g.

**Figure 6:**
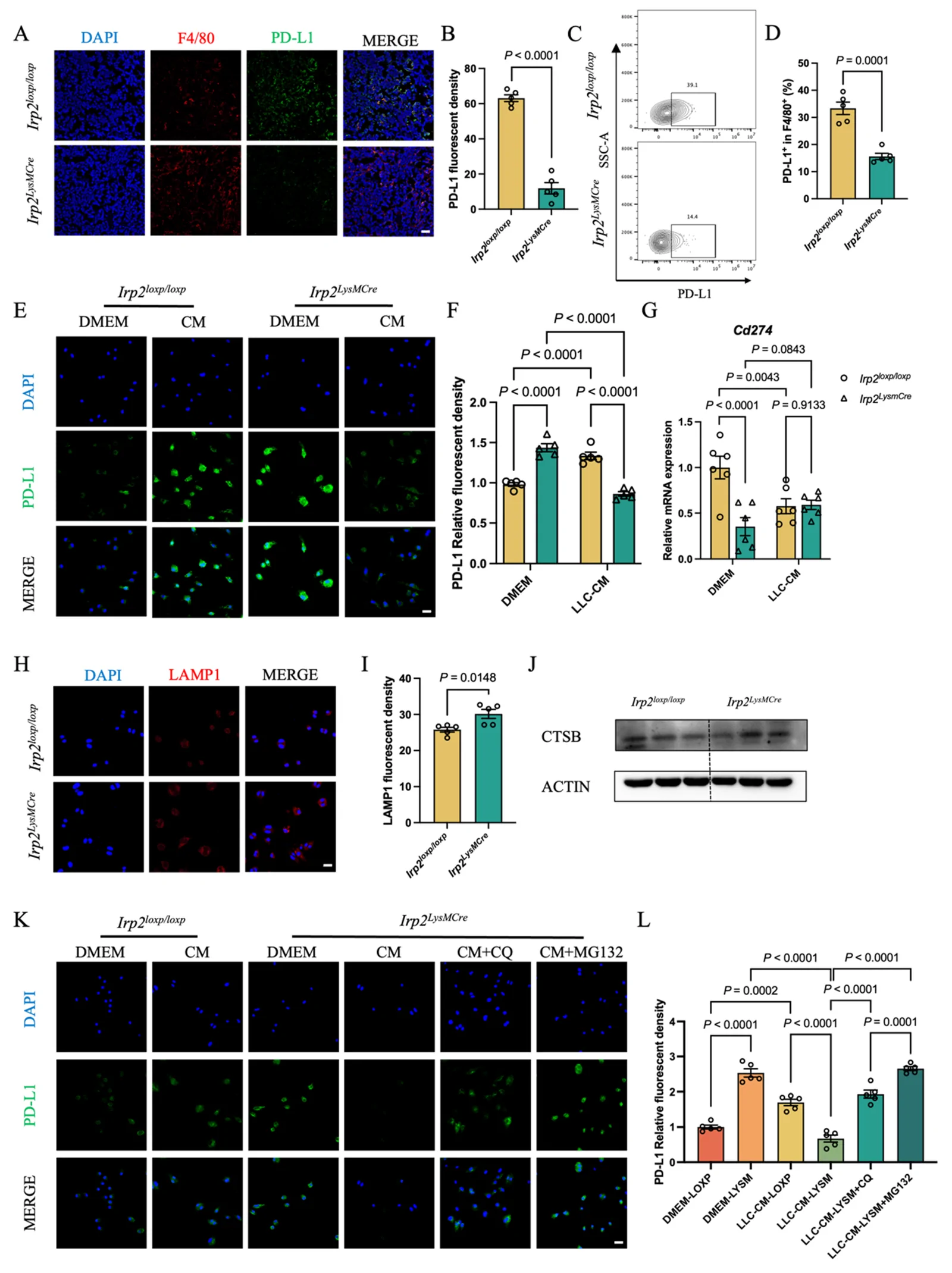
***Irp2* deficiency regulates PD-L1 expression on macrophages.** (A-B) Representative immunofluorescence staining (A) and quantitative analysis (B) of PD-L1 expression on F4/80⁺ macrophages of subcutaneous tumor tissues from *Irp2^loxp/loxp^* and *Irp2^LysMCre^* mice. Scale bar = 30 μm. n = 5. (C-D) Representative flow cytometry plots (C) and quantitative analysis (D) of the percentage of PD-L1⁺ cells in F4/80⁺ macrophages in subcutaneous tumor tissues from *Irp2^loxp/loxp^* and *Irp2^LysMCre^* mice (Hedges' g = -3.938). n = 5. (E-F) Representative immunofluorescence staining (E) and quantitative analysis (F) of membrane PD-L1 expression on *Irp2^loxp/loxp^* and *Irp2^LysMCre^* BMDMs after 48-h culture in DMEM or LLC-CM. Scale bar = 20 μm. n = 5. (G) Quantitative RT-qPCR detection of *Cd274* mRNA levels in *Irp2^loxp/loxp^* and *Irp2^LysMCre^* BMDMs after 48-h culture in DMEM or LLC-CM. n = 6. (H-I) Representative immunofluorescence staining (H) and quantitative analysis (I) of LAMP1 expression in LLC-CM-induced *Irp2^loxp/loxp^* and *Irp2^LysMCre^* TAMs (Hedges' g = 1.767). Scale bar = 20 μm. n = 5. (J) Western blot of CTSB expression in *Irp2^loxp/loxp^* and *Irp2^LysMCre^* TAMs. n = 3. (K-L) Representative immunofluorescence staining (K) and quantitative analysis (L) of membrane PD-L1 expression on *Irp2^loxp/loxp^* and *Irp2^LysMCre^* BMDMs after 48-h culture in DMEM or LLC-CM, with or without inhibition of lysosomal and proteasomal pathways in *Irp2^LysMCre^* TAMs. Scale bar = 20 μm. n = 5. Data are represented as mean ± SEM. *P* values were determined by two-tailed Student's t-test, one-way or two-way ANOVA. Statistical significance was defined as *P* < 0.05. Effect size was calculated as Hedges' g.

**Figure 7:**
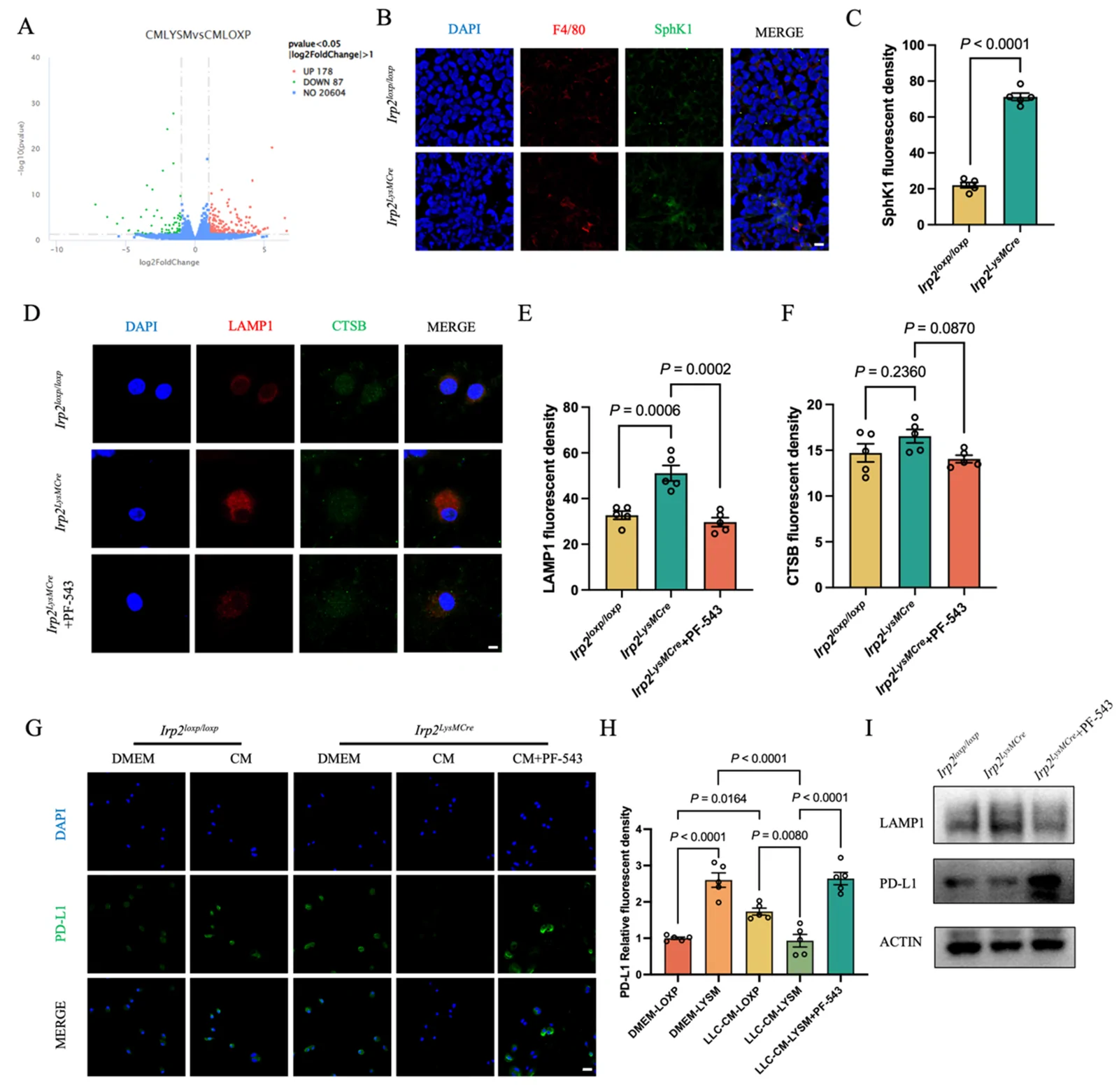
** Identification of SphK1 as a differentially expressed gene in *Irp2*-deficient TAMs and its role in regulating PD-L1 levels.** (A) Volcano plot of differentially expressed genes in* Irp2^loxp/loxp^* and *Irp2^LysMCre^* TAMs. (B-C) Representative immunofluorescence staining (B) and corresponding quantitative analysis (C) of SphK1 expression in F4/80⁺ macrophages in subcutaneous tumor tissues from *Irp2^loxp/loxp^* and *Irp2^LysMCre^* mice. Scale bar = 10 μm. n = 5. (D-F) Representative immunofluorescence staining (D) and quantitative analysis of LAMP1 (E) and CTSB expression in *Irp2^loxp/loxp^* and *Irp2^LysMCre^* TAMs after 48 h of LLC-CM treatment, with or without PF-543. Scale bar = 5 μm. n = 5. (G-H) Representative immunofluorescence staining (G) and corresponding analysis (H) of membrane PD-L1 expression on *Irp2^loxp/loxp^* and *Irp2^LysMCre^* BMDMs after 48 h of treatment with DMEM or LLC-CM, with or without SphK1 inhibition. Scale bar = 20 μm. n = 5. (I) Western blot of LAMP1 and PD-L1 in *Irp2^loxp/loxp^*, *Irp2^LysMCre^* and PF-543-treated* Irp2^LysMCre^* TAMs. Data are represented as mean ± SEM. *P* values were determined by two-tailed Student's t-test or one-way ANOVA. Statistical significance was defined as *P* < 0.05.

**Figure 8:**
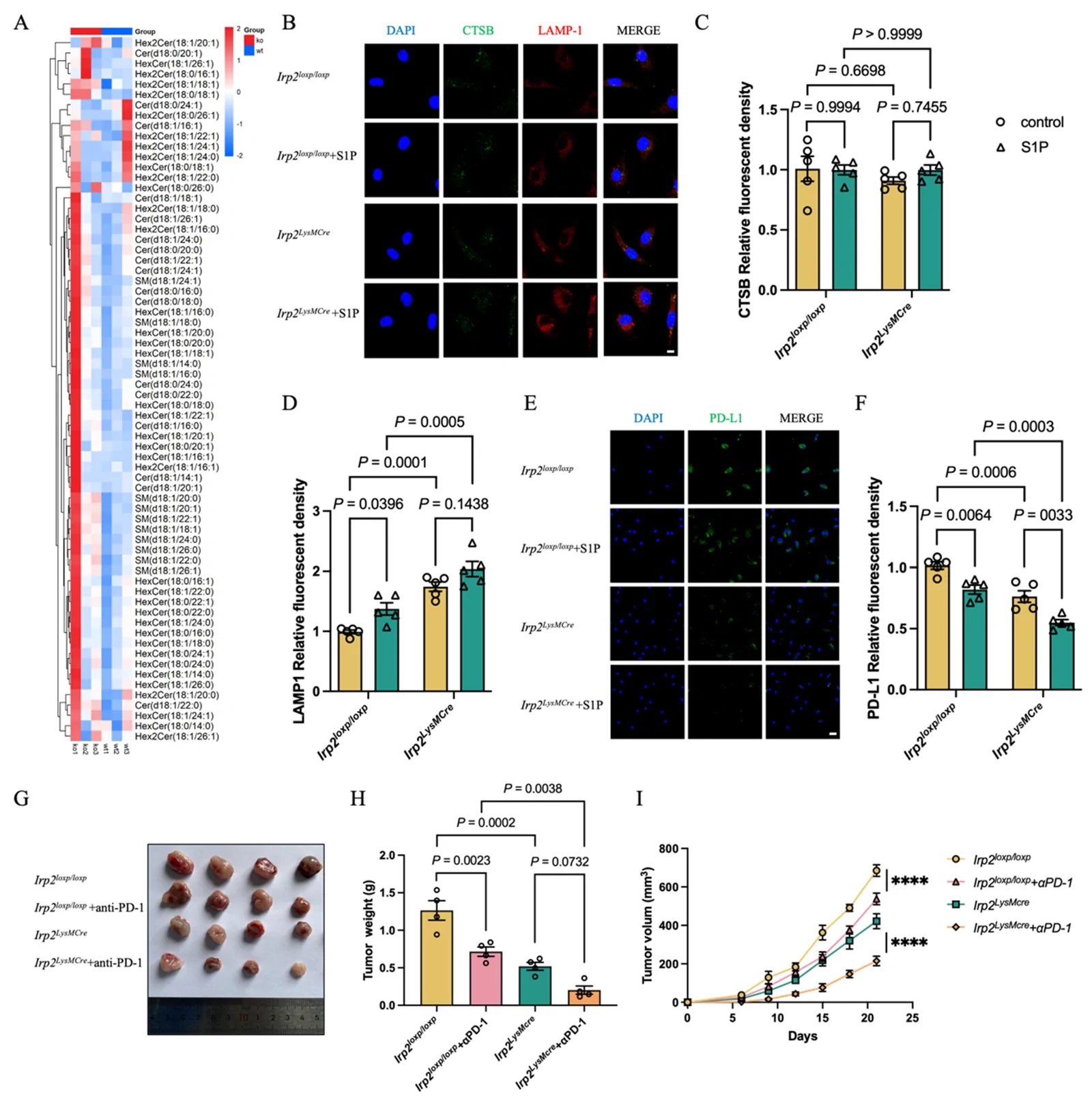
*** Irp2* deficiency remodels sphingolipid metabolism and enhances the response to anti-PD-1 therapy in NSCLC.** (A) Heatmap showing the relative abundance of differentially altered sphingolipid metabolism-related lipid species in LLC-CM-induced* Irp2^loxp/loxp^* and *Irp2^LysMCre^* TAMs. n = 3. (B-D) Representative immunofluorescence staining (B) and the corresponding quantitative analysis of CTSB (C) and LAMP1 (D) in *Irp2^loxp/loxp^* and *Irp2^LysMCre^* TAMs after 48 h of culture in LLC-CM with or without S1P. Scale bar = 5 μm. n =5. (E-F) Representative immunofluorescence staining (E) and corresponding quantitative analysis (F) of PD-L1 in *Irp2^loxp/loxp^* and *Irp2^LysMCre^* TAMs after 48 h of culture in LLC-CM with or without S1P. Scale bar = 20 μm. n =5. (G) Images of subcutaneous tumors in* Irp2^loxp/loxp^* and *Irp2^LysMCre^* mice injected with PBS or anti-PD-1 monoclonal antibody. (H) Subcutaneous tumor weight. (I) Tumor growth curves. n=4. Data are represented as mean ± SEM. *P* values were determined by one-way ANOVA or two-way ANOVA, as appropriate. Statistical significance was defined as *P* < 0.05.

**Figure 9:**
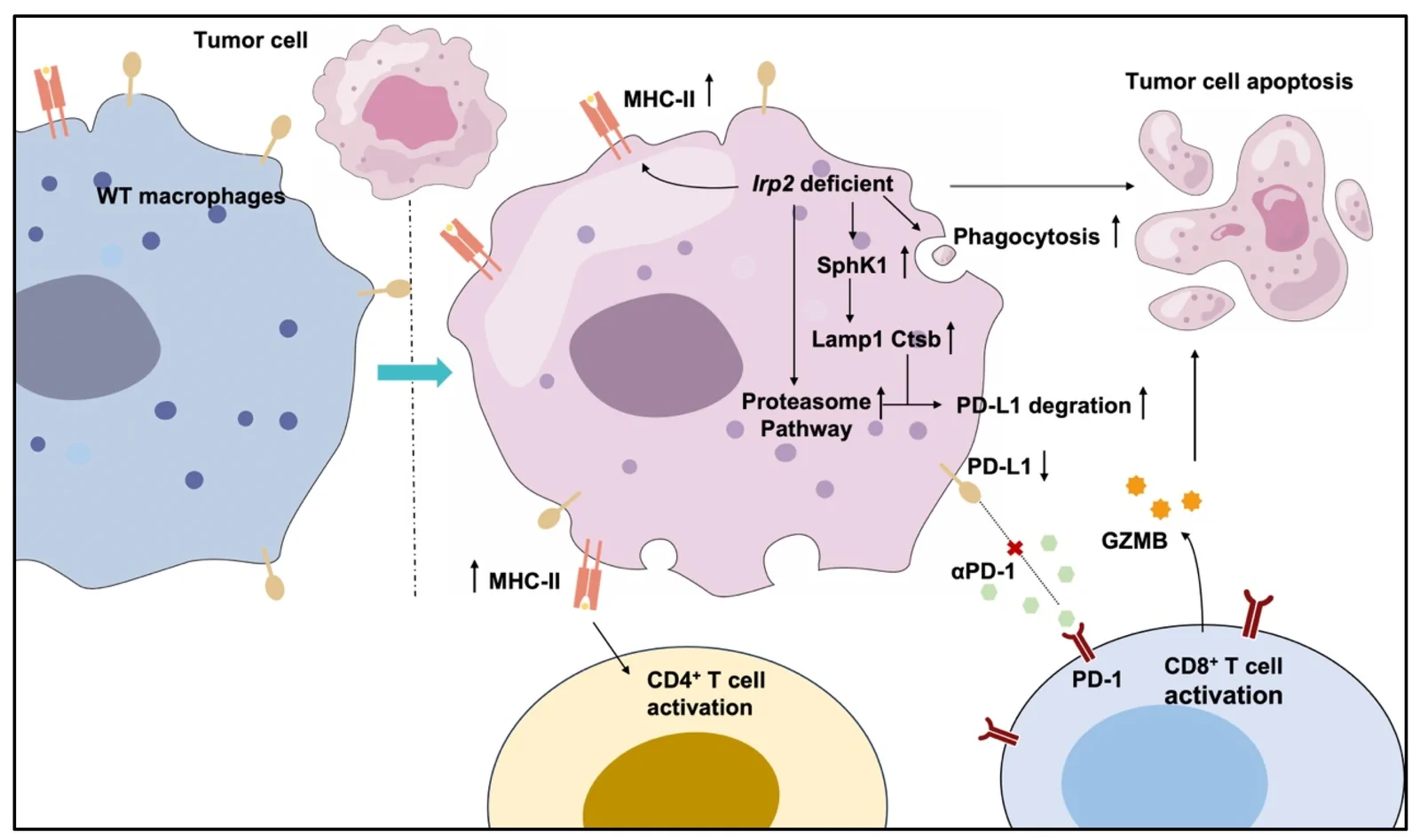
** The molecular mechanism underlying the effects of IRP2 in TAMs.** *Irp2* deficiency promotes the polarization of TAMs toward an M1-like phenotype and enhances their antigen-presenting capacity. *Irp2* deficiency is associated with upregulated SphK1 expression and enhanced macrophage lysosomal function, which may facilitate PD-L1 degradation. Proteasome-associated PD-L1 turnover may also contribute to the reduction in PD-L1 levels. These mechanisms may collectively activate T-cell immune responses, thereby inhibiting tumor progression.
