# Supplementary material for: Targeting Iron Regulatory Protein 2 in Tumor-associated Macrophages Activates Antitumor Immunity in NSCLC via Modulating SphK1-PD-L1 Axis
Source: Int J Biol Sci. 2026 Sep 3;22(14):7928–47. doi: 10.7150/ijbs.130591 (PMC13618223; doi:10.7150/ijbs.130591)
Supplement: Supplementary file 1 [file ijbsv22p7928s1.pdf]

Supplementary Materials

Table S1. Antibodies for Western blot, immunohistochemistry, and immunofluorescence.

Antibody	Cat. No.	Source
IREB2 Rabbit Polyclonal Antibody	R1706-12	HUABIO
anti-CD68 antibody	#ab955	abcam
Pan-Keratin (C11) Mouse mAb	cat#4545	Cell Signaling Technology
Ki-67 Polyclonal antibody	27309-1-AP	Proteintech
Caspase3 antibody	cat#9662S	Cell Signaling Technology
anti-rabbit IgG (HRP)	cat#111-035-144	Jackson ImmunoResearch
anti-mouse IgG (HRP)	cat#115-035-146	Jackson ImmunoResearch
Anti-F4/80 antibody	ab6640	abcam
Anti-F4/80 antibody	ab300421	abcam
PD-L1/CD274 Monoclonal antibody	66248-1-Ig	Proteintech
CD8a Polyclonal antibody	29896-1-AP	Proteintech
Anti-LAMP1 antibody	ab208943	abcam
Cathepsin B Polyclonal antibody	12216-1-AP	Proteintech
CD8 alpha (D4W2Z) Rabbit Monoclonal Antibody	cat#98941	Cell Signaling Technology
CD4 (D7D2Z) Rabbit Monoclonal Antibody	cat#25229	Cell Signaling Technology
granzyme B Antibody (2C5)	sc-8022	Santa Cruz
LAMP1 Recombinant Rabbit	HA722827	HUABIO

Monoclonal Antibody		
SPHK1 Recombinant Rabbit Monoclonal Antibody	ET1704-76	HUABIO
Beta Actin Monoclonal antibody	66009-1-Ig	Proteintech

Table S2. Flow cytometry antibodies.

Antibodies	Catalog	Source	Clone
Zombie NIR Fixable Viability Kit	423105	Biolegend	/
FITC anti-mouse/human CD11b	101205	Biolegend	M1/70
PE/Cyanine7 anti-mouse CD86	105013	Biolegend	GL-1
PE anti-mouse F4/80	123109	Biolegend	BM8
APC anti-mouse CD206	141707	Biolegend	C068C2
Purified Rat Anti-Mouse CD16/CD32	553141	BD Pharmingen	2.4G2
Brilliant Violet 421™ anti-mouse I-A/I-E	107631	Biolegend	M5/114.15.2
PE/Cyanine7 anti-mouse CD4	100527	Biolegend	RM4-5
FITC anti-mouse CD45	103107	Biolegend	30-F11
Brilliant Violet 510 anti-mouse CD8a	100751	Biolegend	53-6.7
PerCP/Cyanine5.5 anti-mouse CD3	100217	Biolegend	17A2
Brilliant Violet 605 anti-mouse CD274 (B7-H1, PD-L1)	124321	Biolegend	10F.9G2

Table S3. Flow cytometry panels.

**Cell Subsets**	**Fluorescence**

	FITC	PE	PerCP	PC7	APC-A750	Violet610	KO525	PB450	APC
Mouse macrophages	CD11b	F4/80	-	CD86	Zombie NIR	PD-L1	-	I-A/I-E	CD206
Mouse T lymphocytes	CD45	-	CD3	CD4	Zombie NIR	-	CD8	-	-

Table S4. Sequences of the primers.

Gene	(5’ – 3’)
Rn18s-Forward Primer	GGCTACCACATCCAAGGAA
Rn18s-Reverse Primer	GCTGGAATTACCGCGGCT
Sphk1-Forward Primer	ATGGAACCAGTAGAATGCCCT
Sphk1-Reverse Primer	TCCGTTCGGTGAGTATCAGTTTA
Il6-Forward Primer	AGTTGCCTTCTTGGGACTGA
Il6-Reverse Primer	GCCACTCCTTCTGTGACTCC
Tnf-Forward Primer	ACGGCATGGATCTCAAAGAC
Tnf-Reverse Primer	GGTCACTGTCCCAGCATCTT
Ym1-Forward Primer	AGGACTCCTGGCTTACTATGA
Ym1-Reverse Primer	AACCAACCCACTCATTACCC
Arg1-Forward Primer	AACCATCTGGGGCATCACAG
Arg1-Reverse Primer	ACCAGAAAGGAACTGCTGGG
Nos2-Forward Primer	ACCTACCGCACCCGAGATG
Nos2-Reverse Primer	AAGCCACTGACACTTCGCACA
Cd80-Forward Primer	CCTCAAGTTTCCATGTCCAAGGC

Cd80-Reverse Primer	GAGGAGAGTTGTAACGGCAAGG
Cd86-Forward Primer	ACGTATTGGAAGGAGATTACAGCT
Cd86-Reverse Primer	TCTGTCAGCGTTACTATCCCGC
Cd74-Forward Primer	GCTGGATGAAGCAGTGGCTCTT
Cd74-Reverse Primer	GATGTGGCTGACTTCTTCCTGG
Cd274-Forward Primer	GGAATTGTCTCAGAATGGTC
Cd274-Reverse Primer	GTAGTTGCTTCTAGGAAGGAG

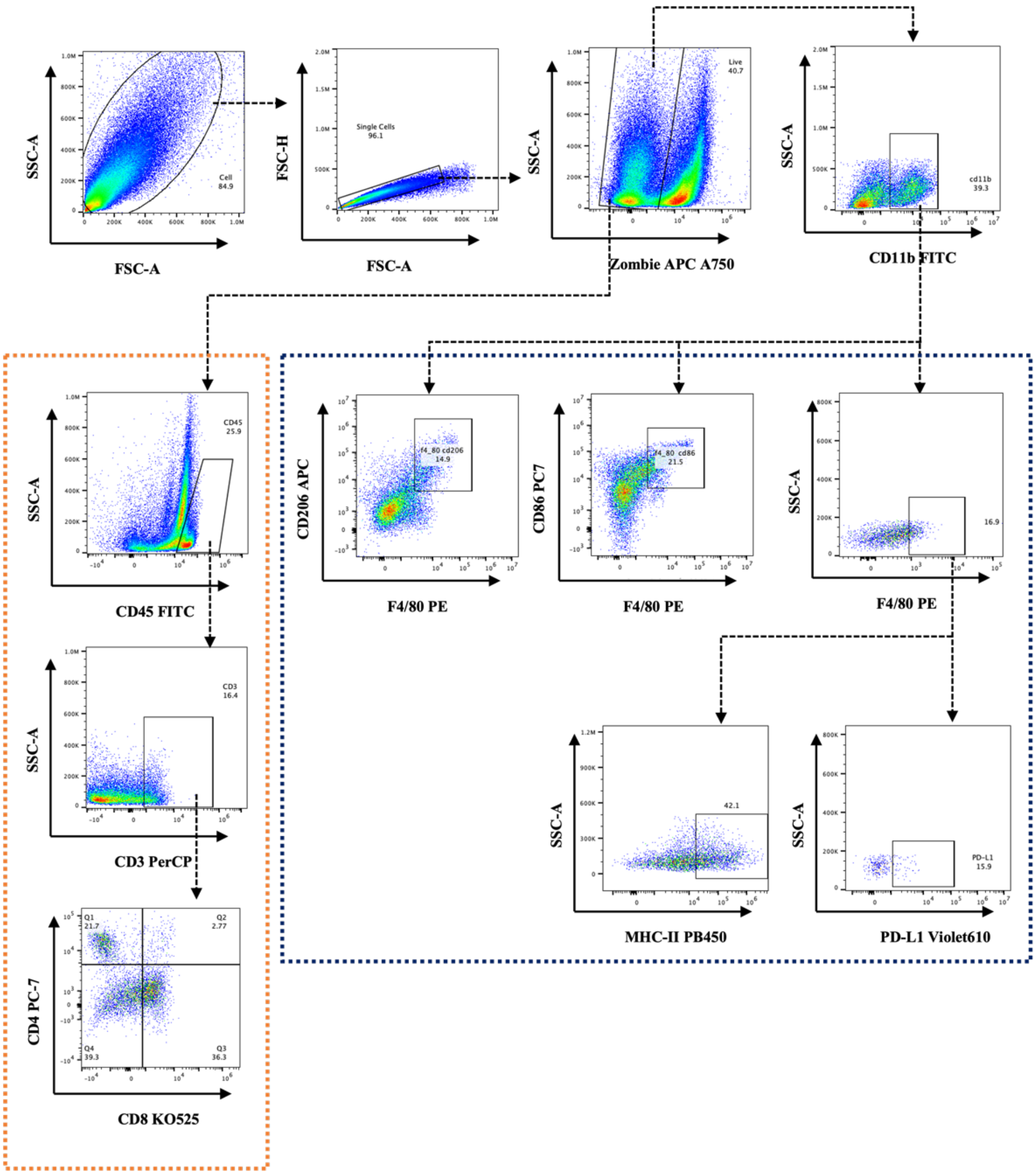

Figure S1. Representative flow cytometry gating strategies used for the analysis of

tumor-infiltrating immune cells.

Cells were first gated according to FSC-A and SSC-A to exclude debris, followed by doublet exclusion using FSC-A versus FSC-H gating. Dead cells were excluded using Zombie NIR fixable viability dye. Tumor-associated macrophages (TAMs) were defined as $CD11b^+F4/80^+$ cells. Within the TAM population, M1-like TAMs were identified based on CD86 expression, whereas M2-like TAMs were identified based on CD206 expression. PD-L1 and MHC-II expression was analyzed within the $CD11b^+F4/80^+$ TAM population. $CD4^+$ T lymphocytes were gated as $CD45^+CD3^+CD4^+$ cells, and $CD8^+$ T lymphocytes were gated as $CD45^+CD3^+CD8^+$ cells. Unstained samples and single-stained compensation controls were used to adjust voltages and set fluorescence compensation. FMO controls were used where appropriate to define positive gates, particularly for CD206, MHC-II, and PD-L1. The same gating strategy was applied consistently to all experimental groups within each corresponding experimental panel.

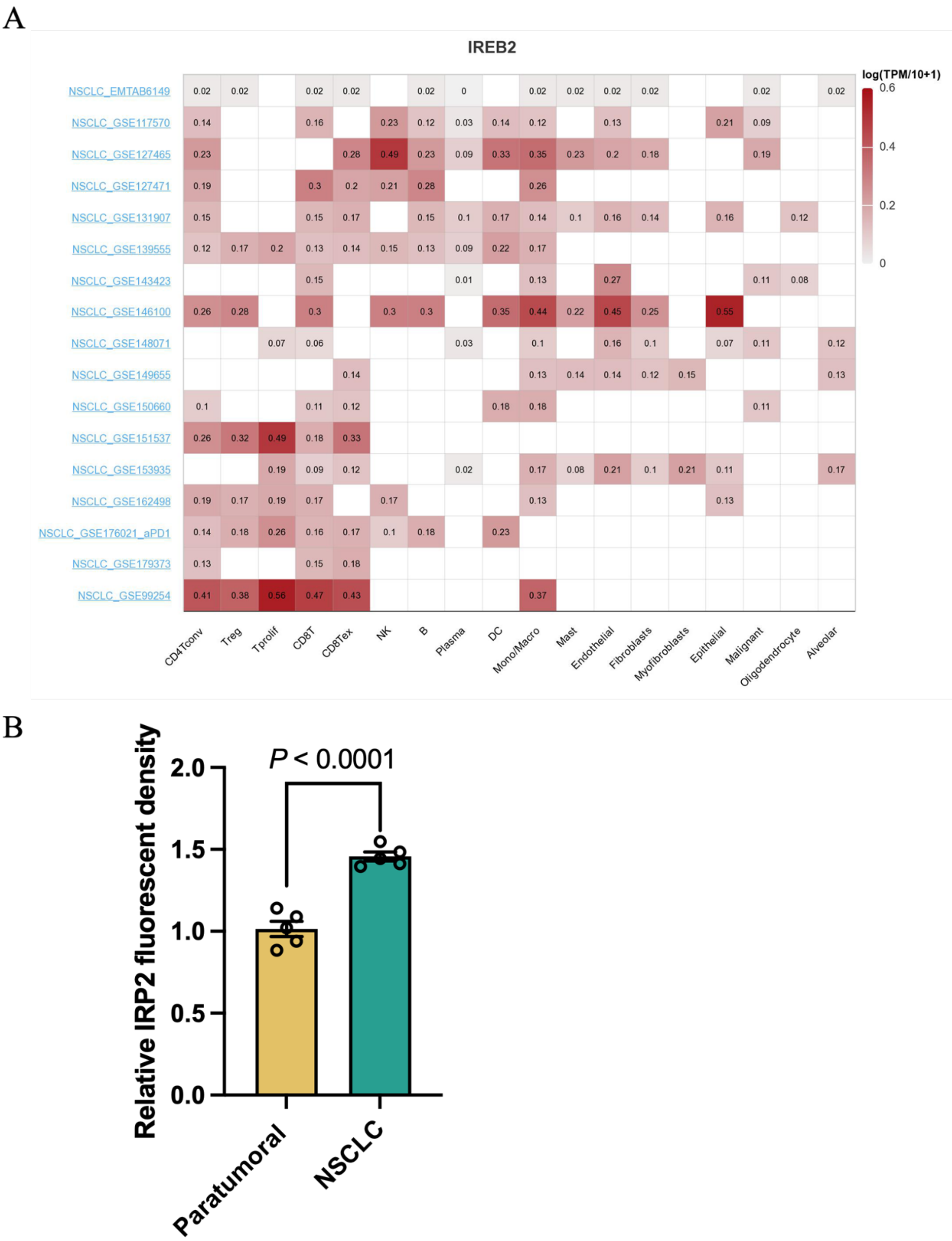

Figure S2. IRP2 distribution and TAM-associated expression in the NSCLC tumor microenvironment.

(A) Analysis of IRP2 cellular distribution in the TME of NSCLC using the TISCH2 database. (B) Quantitative analysis of IRP2 expression in macrophages within NSCLC tumor tissues and adjacent non-tumor tissues. n = 5. Data are represented as mean ±

SEM. P values were determined by two-tailed Student's t-test. Statistical significance was defined as $P < 0.05$.

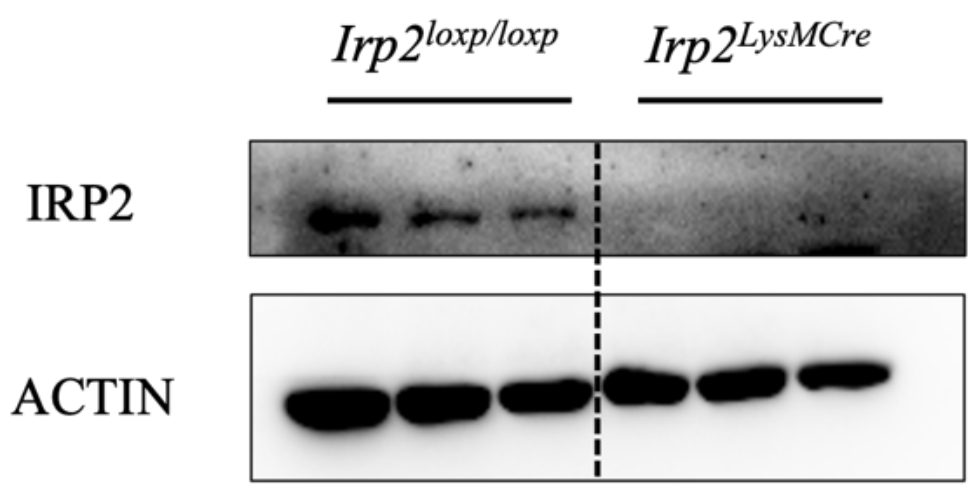

Figure S3. Validation of *Irp2* deletion in macrophages.

Western blot analysis of IRP2 expression in macrophages from *Irp2*$^{loxp/loxp}$ and *Irp2*^{LysMCre} mice. n = 3.

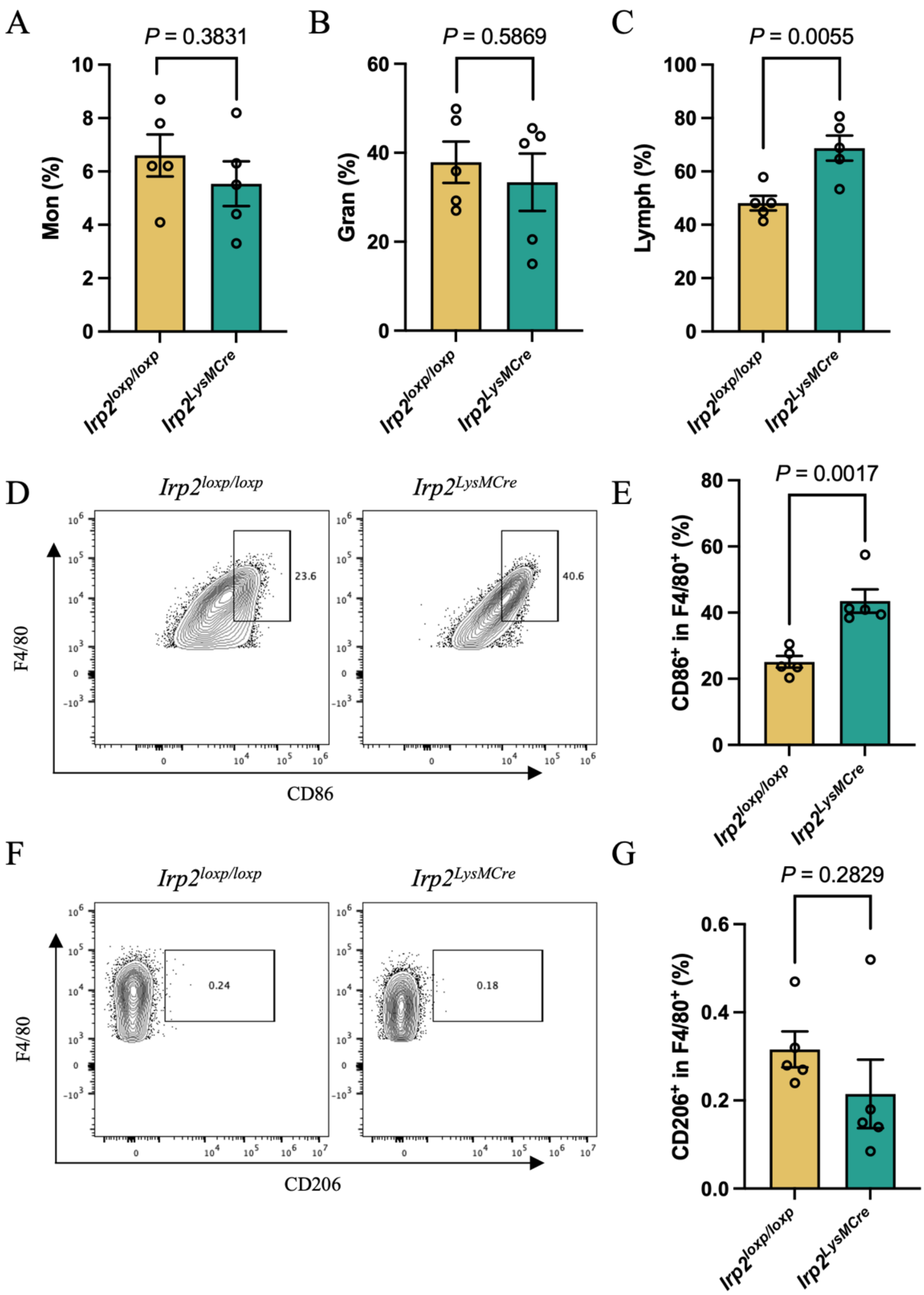

Figure S4. Peripheral immune cell composition and macrophage polarization in LLC-CM-induced TAMs.

(A-C) Percentages of monocytes (A), granulocytes (B) and lymphocytes (C; Hedges' g = 2.153) in peripheral blood routine tests of *Irp2*$^{loxp/loxp}$ and *Irp2*^{LysMCre} mice after subcutaneous LLC tumor modeling. n = 5. (D-E) Representative flow cytometry plots

(D) and quantitative analysis (E) of the proportions of CD86$^+$ cells among F4/80$^+$ LLC-CM-induced $Irp2^{loxp/loxp}$ and $Irp2^{LysMCre}$ TAMs (Hedges' g = 2.648). (F-G) Representative flow cytometry plots (F) and quantitative analysis (G) of the proportions of CD206$^+$ cells among F4/80$^+$ LLC-CM-induced $Irp2^{loxp/loxp}$ and $Irp2^{LysMCre}$ TAMs. n = 5. Data are represented as mean ± SEM. *P* values were determined by two-tailed Student’s t-test. Effect size was calculated as Hedges’ g.

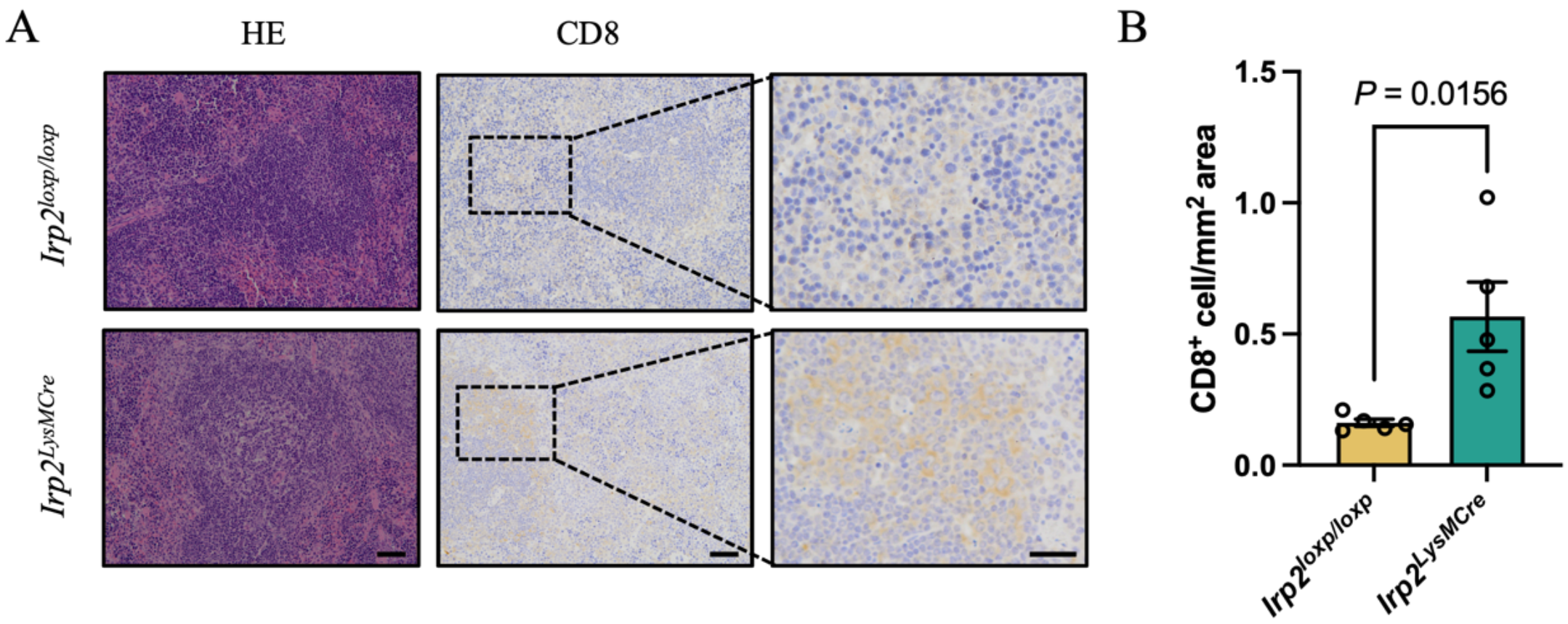

Figure S5. Immunohistochemical analysis of CD8$^+$ T cells in the Spleen of LLC tumor-bearing mice.

(A) H&E staining and CD8 immunohistochemical staining of spleen tissues from $Irp2^{loxp/loxp}$ and $Irp2^{LysMCre}$ mice after subcutaneous LLC tumor modeling. (B) Quantitative analysis of the infiltration percentage of CD8$^+$ T cells in spleen tissues (Hedges' g = 1.748). Scale bar = 50 μm. Scale bars in enlarged images = 25 μm. n = 5. Data are represented as mean ± SEM. *P* values were determined by two-tailed Student’s t-test. Statistical significance was defined as $P < 0.05$. Effect size was calculated as Hedges’ g.

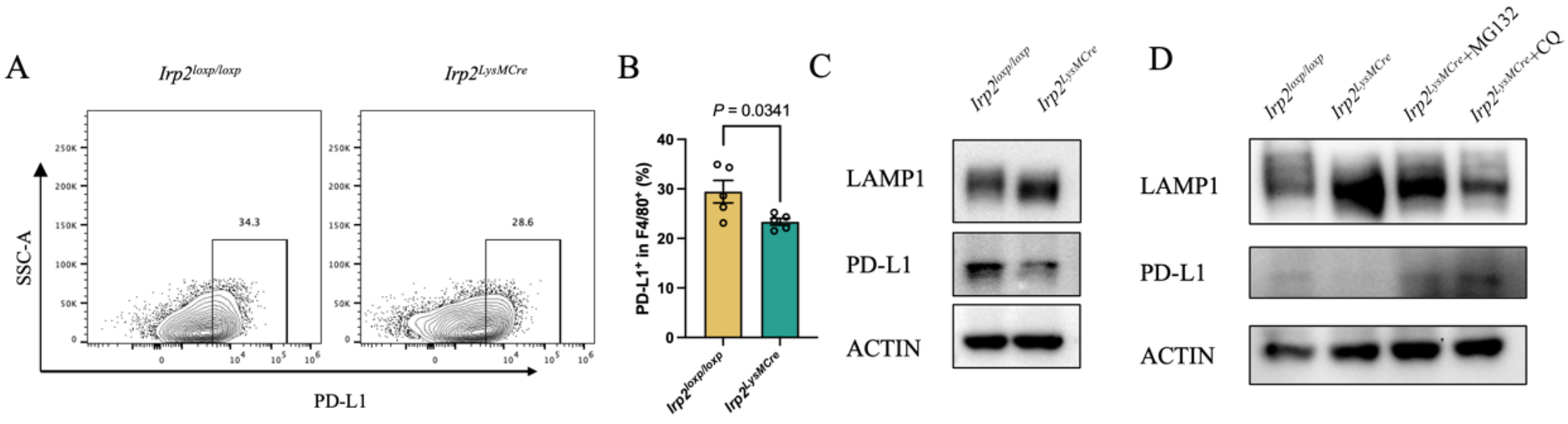

Figure S6. *Irp2* deficiency regulates PD-L1 expression and PD-L1 degradation in TAMs.

(A-B) Representative flow cytometry plots (A) and quantitative analysis (B) of the percentage of PD-L1^{+} cells in F4/80^{+} LLC-CM-induced *Irp2*$^{loxp/loxp}$ and *Irp2*^{LysMCre} TAMs (Hedges' g = -1.457). n=5. (C) Western blot of PD-L1 and LAMP1 expression in LLC-CM-induced *Irp2*$^{loxp/loxp}$ and *Irp2*^{LysMCre} TAMs. (D) Western blot of PD-L1 and LAMP1 expression in LLC-CM-induced *Irp2*$^{loxp/loxp}$, *Irp2*^{LysMCre} TAMs and *Irp2*^{LysMCre} TAMs treated with the lysosomal inhibitor chloroquine (CQ) or the proteasome inhibitor MG132. Data are represented as mean ± SEM. *P* values were determined by two-tailed Student's t-test. Statistical significance was defined as $P < 0.05$. Effect size was calculated as Hedges' g.

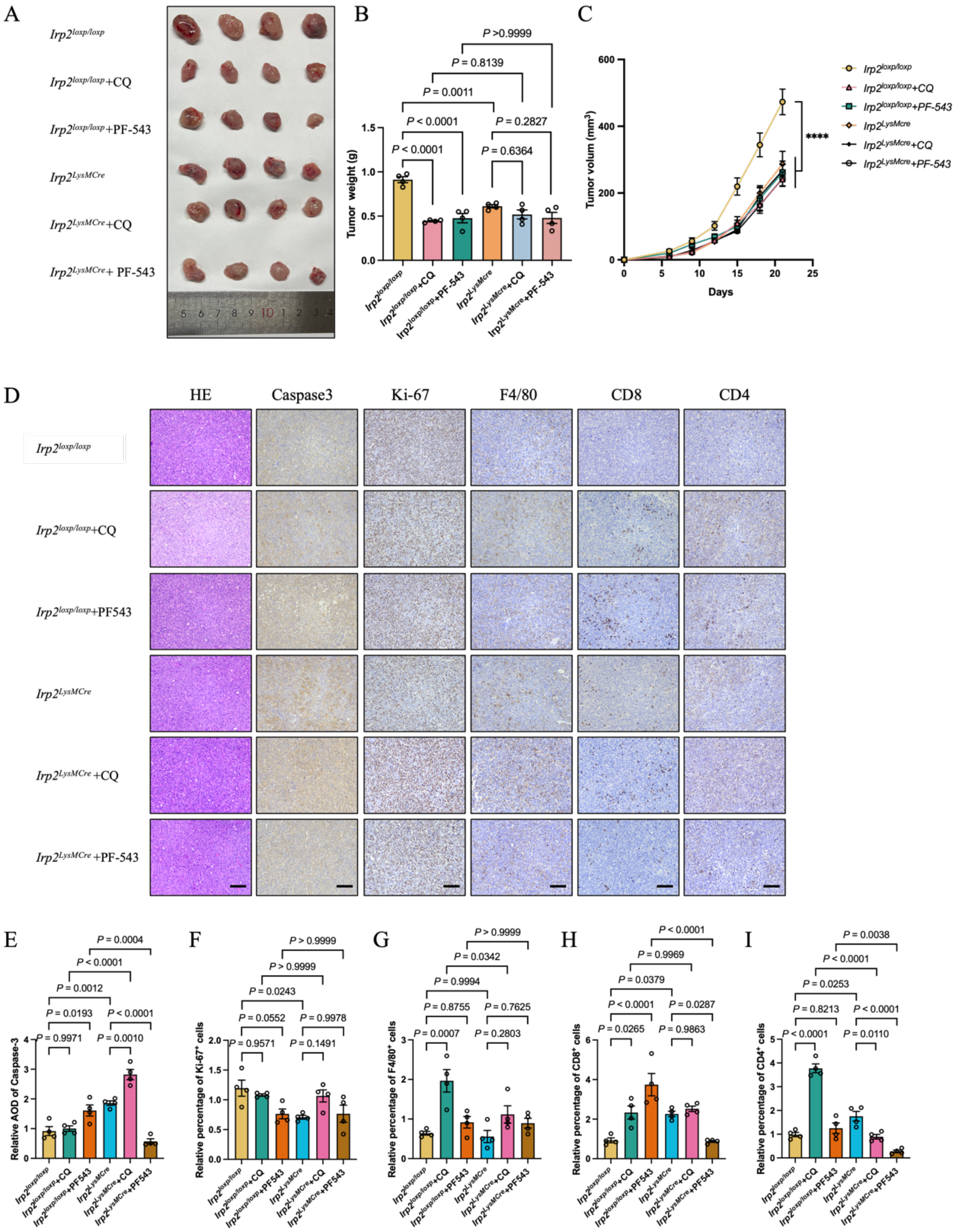

Figure S7. In vivo inhibition of lysosomal activity and SphK1 modulates IRP2-associated antitumor regulation in TAMs.

(A) Representative images of subcutaneous LLC tumors from *Irp2*$^{loxp/loxp}$ and *Irp2*^{LysMCre} mice after treatment with CQ or PF-543. (B) Tumor weight of *Irp2*$^{loxp/loxp}$ and *Irp2*^{LysMCre} mice after CQ or PF-543 treatment. (C) Tumor growth curves of

Irp2$^{loxp/loxp}$ and *Irp2*^{LysMCre} mice after CQ or PF-543 treatment. (D) Representative H&E staining and IHC staining of Caspase-3, Ki-67, F4/80, CD8, and CD4 in subcutaneous tumor tissues. (E-I) Quantitative analysis of Caspase-3 expression (E), infiltration of Ki-67^{+} (F), F4/80^{+} (G), CD8^{+} (H), and CD4^{+} (I) in tumor tissues. Scale bar = 50 μm. n = 4. Data are represented as mean ± SEM. *P* values were determined by one-way ANOVA or two-way ANOVA, as appropriate.

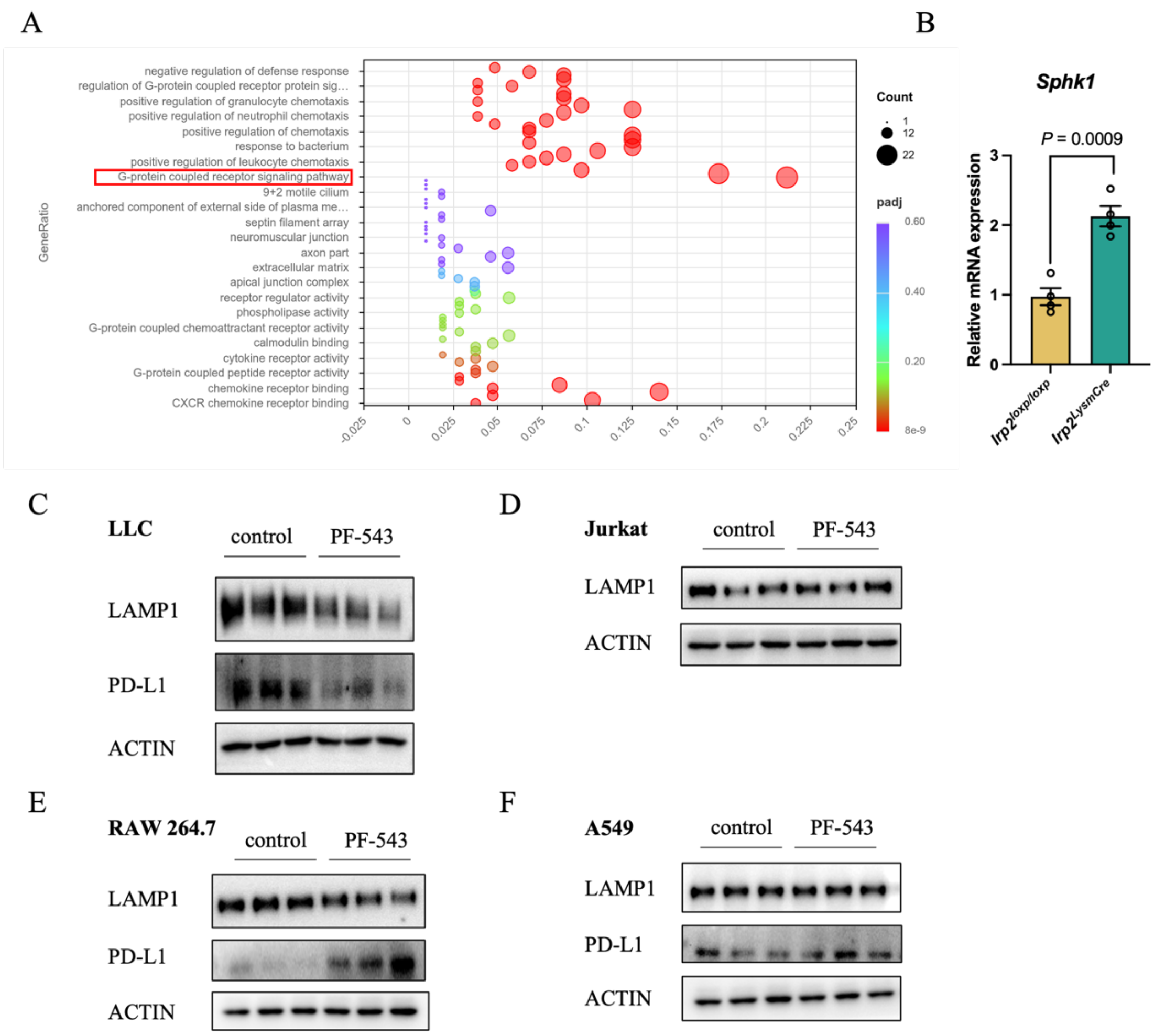

Figure S8. ***Irp2*** **deficiency activates SphK1-associated signaling and PF-543 modulates LAMP1 and PD-L1 expression in multiple cell types.**

(A) GO functional analysis showing the pathways enriched among genes upregulated in RNA-sequencing of *Irp2*$^{loxp/loxp}$ and *Irp2*^{LysMCre} TAMs. (B) RT-qPCR detection of the mRNA expression levels of *Sphk1* in *Irp2*$^{loxp/loxp}$ and *Irp2*^{LysMCre} TAMs after 48 h of induction with LLC-CM (Hedges' g = 3.732). n = 4. (C-F) Western blot of LAMP1 and PD-L1 expression in LLC (C), RAW 264.7 (E) and A549 (F) cells, and of LAMP1 expression in Jurkat cells (D), following treatment with PF-543 for 4 h. n = 3. Data are represented as mean ± SEM. *P* values were determined by two-tailed Student's t-test. Statistical significance was defined as $P < 0.05$. Effect size was calculated as Hedges'

g.

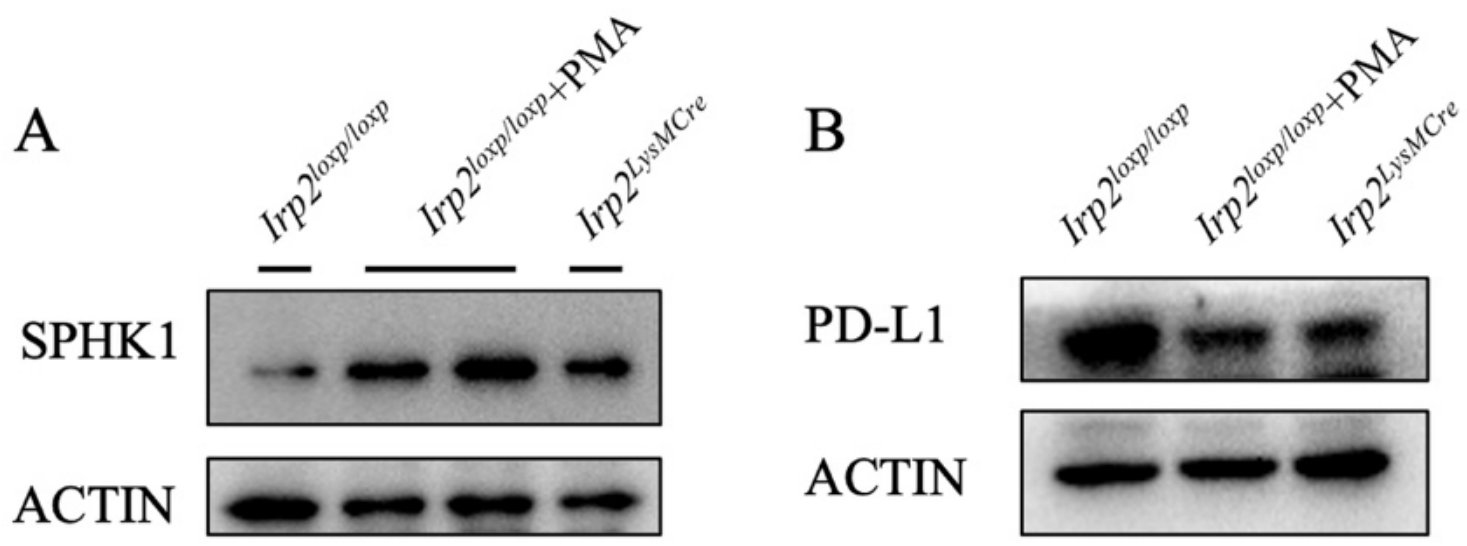

Figure S9. Effects of PMA-induced SphK1 upregulation on PD-L1 expression in *Irp2*$^{loxp/loxp}$ TAMs.

(A) Western blot analysis of SphK1 expression in *Irp2*$^{loxp/loxp}$ TAMs, PMA-treated *Irp2*$^{loxp/loxp}$ TAMs, and *Irp2*^{LysMCre} TAMs. (B) Western blot analysis of PD-L1 expression in *Irp2*$^{loxp/loxp}$ TAMs, PMA-treated *Irp2*$^{loxp/loxp}$ TAMs, and *Irp2*^{LysMCre} TAMs.
